# Life experiences of humiliation, entrapment, and frontoparietal-cerebellar connectivity predict adolescent anxiety and depression symptoms

**DOI:** 10.1017/S0033291726103699

**Published:** 2026-03-30

**Authors:** Yueyue Lydia Qu, Sidhant Chopra, Shijie Qu, Carrisa V. Cocuzza, Loïc Labache, Clemens C.C. Bauer, Francesca Morfini, Susan Whitfield-Gabrieli, George M. Slavich, Jutta Joormann, Avram J. Holmes

**Affiliations:** 1 Department of Psychology, Yale University, New Haven, USA; 2 Wu Tsai Institute, Yale University, New Haven, USA; 3 Orygen, The University of Melbourne, Melbourne, Australia; 4 Centre for Youth Mental Health, The University of Melbourne, Melbourne, Australia; 5 Department of Psychiatry, Brain Health Institute, Rutgers University, Piscataway, USA; 6 Department of Psychology, Northeastern University, Boston, USA; 7 Center for Cognitive & Brain Health, Northeastern University, Boston, USA; 8 Department of Brain and Cognitive Sciences and McGovern Institute for Brain Research, Massachusetts Institute of Technology, Cambridge, USA; 9 Center for Depression, Anxiety and Stress Research, McLean Hospital, Belmont, USA; 10 Department of Psychiatry, Harvard Medical School, Boston, USA; 11 Department of Psychiatry and Biobehavioral Sciences, University of California, Los Angeles, Los Angeles, USA

**Keywords:** adolescence, anxiety, depression, longitudinal prediction, major life stressor characteristics, resting-state functional networks

## Abstract

**Background:**

Exposure to major life stressors and aberrant brain functioning have been related to anxiety and depression in adolescence. However, whether these associations differ based on the specific characteristics of the stressors and/or brain functional networks remains unclear.

**Methods:**

In a longitudinal sample of adolescents enriched for anxiety and depressive disorders, we examined cumulative lifetime stressor frequency and severity of five stressor characteristics: physical danger, interpersonal loss, humiliation, entrapment, and role change/disruption. Anxiety and depression symptoms were assessed at three time points: baseline, 6-month, and 12-month follow-ups. Linear mixed-effect models tested if lifetime frequency and severity of these stressor characteristics were associated with anxiety and depression symptoms across the three time points. We also identified whole-brain resting-state functional connectivity (RSFC) features linked to the predictive stressor characteristics and examined their associations with anxiety and depression symptoms across time.

**Results:**

Controlling for all other stressor characteristics and covariates, lifetime frequency and severity of humiliation and entrapment predicted greater anxiety and depression symptoms across the three time points. After additionally accounting for co-occurring depression and anxiety symptoms, only entrapment frequency and severity remained significant. More negative RSFC between the frontoparietal network and the left cerebellum were linked to greater entrapment severity at baseline, and associated with greater anxiety and depression symptoms across time.

**Conclusions:**

Our study highlights lifetime exposures to humiliation and entrapment as central stressor characteristics linked to adolescent internalizing symptoms. Our results also suggest that frontoparietal–left cerebellar connectivity may be a stress-sensitive marker of adolescent internalizing problems over time.

## Introduction

Exposure to major life stressors is a strong risk factor for the onset and subsequent recurrence of affective disorders (Faravelli & Pallanti, 1989; Francis, Moitra, Dyck, & Keller, 2012; Kendler, Karkowski, & Prescott, 1999; Kessler, 1997; Miloyan, Bienvenu, Brilot, & Eaton, 2018), especially in adolescence when there is increased brain plasticity and heightened vulnerability to the emergence of psychopathology (Casey, Jones, & Hare, 2008; March-Llanes et al., 2017; Merikangas et al., 2010, 2010; Paus, Keshavan, & Giedd, 2008). However, stressors come in many different forms and are diverse with respect to both their characteristics and associations with mental health outcomes (Bonanno, Chen, & Galatzer-Levy, 2023; Cohen, Murphy, & Prather, 2019; A. H. Lee et al., 2025; Serafini et al., 2023; Slavich, 2019). This has wide-ranging implications for youth mental health problems, which may increase the risk of suicidal behavior (Bertuccio et al., 2024). Results from large-scale, prospective cohort studies have suggested that exposures to certain characteristics of major life stressors may preferentially increase risk for specific clinical outcomes, such as anxiety and depression. For instance, stressor characteristics that involve devaluation of the self, such as interpersonal loss and humiliation, are theorized to preferentially heighten risk for depression (Asselmann et al., 2015; Farmer & McGuffin, 2003; Finlay-Jones & Brown, 1981; Keller, Neale, & Kendler, 2007; Kendler et al., 2003). In contrast, stressor characteristics marked by a threat to one’s physical integrity, such as danger, are theorized to be stronger predictors of anxiety (Asselmann et al., 2015; Ayazi et al., 2014; Finlay-Jones & Brown, 1981; Kendler et al., 2003). Stressors characterized by feelings of failure without any means of escape, such as entrapment, predict both anxiety and depression (Griffiths et al., 2014; Kendler et al., 2003). Although distinct stressor characteristics are not mutually exclusive from each other (e.g. a stressor can be both dangerous and humiliating), prior studies have often examined each stressor characteristic in isolation. Therefore, the *unique contributions* of each stressor characteristic to adolescent anxiety and depression symptoms remain unclear.

Aside from exposures to major life stressors, seed- and network-based resting-state functional connectivity (RSFC) patterns have similarly been associated with prospective internalizing symptoms in preadolescent children and adolescents (Connolly et al., 2017; Huang et al., 2023; Jalbrzikowski et al., 2017; Jin et al., 2020; Lopez, Luby, Belden, & Barch, 2018; Morfini et al., 2025; Pawlak, Bray, & Kopala-Sibley, 2022; Qu, Rappaport, Luby, & Barch, 2021; Strikwerda-Brown et al., 2014). Other studies extend this line of work to show that changes in cortical–cortical and cortical–subcortical circuits may index pathways through which stress exposure relates to prospective internalizing symptoms in adolescence (Brieant, Sisk, & Gee, 2021; Chahal et al., 2022; Rakesh et al., 2021; Rakesh, Allen, & Whittle, 2023; Tsomokos, Tiemeier, Slavich, & Rakesh, 2025; Xiao et al., 2025; Yang et al., 2024). Yet, most of these studies operationalized stress as a total exposure score, and comparatively few differentiated between specific stressor characteristics (Chahal et al., 2022; Rakesh et al., 2023).

### Present study

To address these gaps in knowledge, we conducted two complementary analyses using linear mixed-effect (LME) models in a longitudinal sample of adolescents recruited from school-based and hospital-based child treatment programs (Hubbard et al., 2024). Most participants had a current diagnosis of at least one anxiety or depressive disorder at the time of baseline assessment. First, we assessed the unique prospective associations of lifetime exposures (self-reported number of exposures [henceforth referred as ‘frequency’] and subjective severity ratings [henceforth referred as ‘severity’]) of five stressor characteristics – physical danger, interpersonal loss, humiliation, entrapment, and role change/disruption – by entering them simultaneously as predictors of anxiety and depression symptoms across baseline, 6-month, and 12-month follow-up assessments. To quantify symptom-specific associations in the context of high comorbidity between anxiety and depression, we additionally adjusted for time-varying co-occurring symptoms at each wave. Second, we identified whole-brain network- and network–subcortical RSFC features, which are associated with the stressor characteristics most predictive of anxiety and depression symptoms from the first set of analyses, and tested whether these RSFC features are associated with anxiety and depression symptoms across baseline, 6-month, and 12-month follow-up assessments.

## Methods and materials

### Participants

Data were collected from 215 adolescents (*M*_age_ = 15.44, range = 14-17 years old) enrolled in the Boston Adolescent Neuroimaging of Depression and Anxiety study (Hubbard et al., 2024) and assessed at 6-month intervals after the initial visit for up to 12 months. The present analyses were restricted to data from the baseline, 6-month, and 12-month follow-up assessments. Resting-state functional magnetic resonance imaging (rsfMRI) data were available for 202 participants at baseline (Hubbard et al., 2024). Out of these 202 adolescents, 150 had available behavioral measures of interest at baseline, 6-month, and 12-month follow-up assessments and were included in the final sample for stress-symptom analyses (Table 1). Of these 150 participants, 130 were included in the final sample for brain-symptom analyses (see **Neuroimaging** for details), and 65% (*n* = 98) had a current diagnosis of at least one anxiety or depressive disorder. The diagnostic group of each participant was given by a blinded, licensed clinical psychologist based on the Diagnostic and Statistical Manual of Mental Health Disorders, 5th Edition (DSM-5; American Psychiatric Association, 2013) and reached moderate to substantial inter-rater agreement (Hubbard et al., 2024).

**Table 1.** Demographic characteristics for the final analytical samples for stress-symptom (*n* = 150) and brain-symptom (*n* = 130) analyses

	Stress-symptom analyses (*n* = 150)		Brain-symptom analyses (*n* = 130)	
Baseline				
Demographics	*M/n*	SD/%	*M/n*	SD/%
Age	15.44	0.85	15.44	0.84
Sex (% female)	92	61.33	81	62.3
Mean parental education^a^	6.11	1.27	6.16	1.27
Diagnostic group	*n*	*%*	*n*	*%*
Anxiety^b^	58	38.67	49	37.69
Control	52	34.67	45	34.62
Depression^c^	40	26.67	36	27.69
Race	*n*	*%*	*n*	*%*
African American	3	2.00	1	0.77
Asian	3	2.00	3	2.31
Hawaiian	1	0.67	1	0.77
White	121	80.67	104	80.00
Ethnicity	*n*	*%*	*n*	*%*
Hispanic	11	7.33	11	8.46
Lifetime frequency of stressor characteristics	*M*	SD	*M*	SD
Entrapment (range: 0–14; possible range: 0–28)	4.36	3.18	4.28	2.98
Humiliation (range: 0–20; possible range: 0–40)	5.85	4.60	5.69	4.43
Interpersonal loss (range: 0–20; possible range: 0–61)	5.08	3.92	5.00	3.83
Physical danger (range: 0–10; possible range: 0–46)	1.98	2.18	1.96	2.16
Role change/disruption (range: 0–15; possible range: 0–24)	2.99	2.47	2.95	2.30
Lifetime severity ratings of stressor characteristics	*M*	SD	*M*	SD
Entrapment (range: 0–54; possible range: 0–95)	13.65	12.01	13.47	11.29
Humiliation (range: 0–36; possible range: 0–60)	8.88	7.92	8.65	7.46
Interpersonal loss (range: 0–45; possible range: 0–85)	12.09	9.57	12.15	9.56
Physical danger (range: 0–38; possible range: 0–60)	5.87	6.81	5.74	6.41
Role change/disruption (range: 0–32; possible range: 0–60)	7.20	6.51	7.19	6.32
Clinical symptoms	*M*	SD	*M*	SD
Anxiety symptoms (range: 0–73; possible range: 0–93)	26.05	18.24	25.53	17.83
Depression symptoms (range: 0–56; possible range: 0–66)	16.84	15.28	16.33	14.61
6-month follow-up				
Clinical symptoms	*M*	SD	*M*	SD
Anxiety symptoms (range: 0–79; possible range: 0–93)	24.86	17.51	24.02	16.19
Depression symptoms (range: 0–58; possible range: 0–66)	17.14	14.60	16.49	13.78
12-month follow-up				
Clinical symptoms	*M*	SD	*M*	SD
Anxiety symptoms (0–93) (range: 0–62; possible range: 0–93)	21.72	15.24	21.48	14.46
Depression symptoms (0–66) (range: 0–53; possible range: 0–66)	14.95	13.90	14.75	13.65

a Mean parental education = the average of the highest education degree achieved across both parents for each subject.

b Anxiety = having a current diagnosis of at least one anxiety disorder and no depressive disorder based on DSM-5.

c Depression = having a current diagnosis of at least one depressive disorder based on DSM-5.

### Measures of social-psychological characteristics of life stressors

At the baseline assessment, the Stress and Adversity Inventory for Adolescents (STRAIN; Slavich, 2019) assessed each participant’s total lifetime frequency and severity ratings of exposures to five social-psychological stressor characteristics (Supplementary Figures 1 and 2) – physical danger, interpersonal loss, humiliation, entrapment, and role change/disruption – by quantifying both the frequency and subjective severity ratings of self-reported acute and chronic stressors across the life course (see https://www.strainsetup.com). Examples of stressors linked to each of these five characteristics have been described elsewhere (Slavich et al., 2019). Self-reported severity ratings are a good complementary measure to frequency of major life stressors because subjective ratings do not assume that all stressors are perceived equally by all individuals and may thus index important individual differences in stress vulnerability (Shields et al., 2023). Brief definitions for these stressor characteristics are described in the supplement (Brown, Harris, & Hepworth, 1995; Finlay-Jones & Brown, 1981; Kendler et al., 2003; Slotter & Walsh, 2017, Supplementary Method 1).

The STRAIN has demonstrated excellent test–retest reliability, good concurrent and discriminant validity, as well as predictive utility in relation to various clinical outcomes, including anxiety and depression (Slavich, 2019; Slavich & Shields, 2018).

### Measures of anxiety and depression symptoms

For each participant, anxiety and depression symptoms were assessed at three time points: baseline, 6-month, and 12-month follow-ups. The mean anxiety and depressive symptoms within each diagnostic group at each of the three time points are illustrated in Supplementary Figure 3.

Self-reported anxiety symptoms were assessed by the Revised Children’s Anxiety and Depression Scale (RCADS; Chorpita et al., 2000). The RCADS has exhibited excellent internal consistency, test–retest reliability, and convergent and discriminant validity (Chorpita et al., 2000). The RCADS has six subscales: separation anxiety disorder, social phobia, generalized anxiety, panic disorder, obsessive-compulsive disorder, and low mood. Total anxiety symptoms for each participant were computed by summing the four anxiety subscales (Separation Anxiety Disorder, Social Phobia, Generalized Anxiety, and Panic Disorder).

Self-reported depression symptoms were assessed by the Mood and Feelings Questionnaire (MFQ; Angold, Costello, Messer, & Pickles, 1995; Costello & Angold, 1988). Prior studies have found the MFQ to be a reliable and valid measure of adolescent depression in both clinical and nonclinical samples across different populations (Burleson Daviss et al., 2006; Sund, Larsson, & Wichstrøm, 2001; Thabrew et al., 2018; Wood, Kroll, Moore, & Harrington, 1995). We used the total MFQ score as the measure of depression symptoms for each participant.

### Neuroimaging

#### Data acquisition and processing

Functional and anatomical neuroimaging data were acquired at baseline assessment using a 3-Tesla Siemens Prisma scanner with a two-dimensional multiband gradient-recalled echo-planar imaging sequence. Each participant underwent four 5.8-minute rsfMRI runs, consisting of two runs with opposite phase encoding directions. Each rsfMRI scan was acquired using 2-mm isotropic resolution and a repetition time (TR) of 800ms. Full details of the acquisition protocol can be found elsewhere (Siless et al., 2020).

The acquired rsfMRI data then went through the previously established Human Connectome Project (HCP) minimal preprocessing pipelines (Glasser et al., 2013). The minimally preprocessed rsfMRI data for each run were then denoised using Independent Components Analysis with FIX (ICA-FIX) (Griffanti et al., 2014; Salimi-Khorshidi et al., 2014) pretrained using HCP_hp2000.RData and aligned across participants using MSMAll multimodal surface registration (Glasser et al., 2016; Robinson et al., 2014). The ICA-FIXed rsfMRI data were further processed by detrending and applying a temporal band-pass filter with a high-pass cutoff of 0.008Hz and a low-pass cutoff of 0.09 Hz. We then regressed out the global signal and 12 motion parameters from the resulting data. Finally, the cortical vertices were smoothed with a Gaussian kernel (2 mm Full Width at Half Maximum). Out of the 150 participants with available behaviors of interest across the three time points, we excluded participants (*n* = 20) if their mean framewise displacement (FD) (Power et al., 2012) was >0.30mm, or if any single volume exceeded 5 mm FD (Parkes, Fulcher, Yücel, & Fornito, 2018; Satterthwaite et al., 2013). Consequently, our final sample for statistical analyses involving RSFC and symptom measures comprised 130 participants for brain-symptom analyses (Table 1).

#### Resting-state functional connectivity

**Figure 1.**
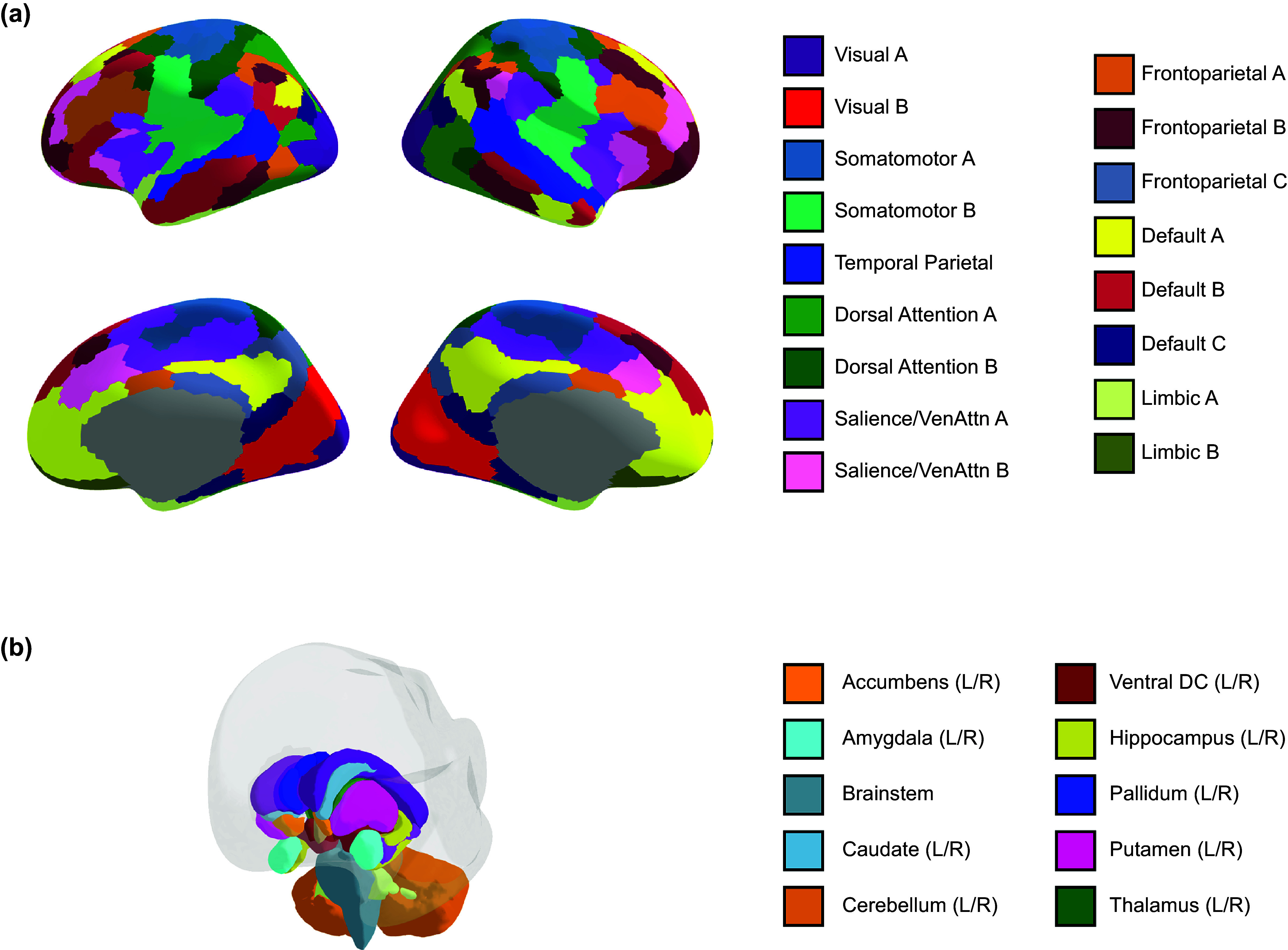
The functional network organization of the human cerebral cortex was revealed through intrinsic functional connectivity. Colors reflect regions estimated to be within the same network. (a) Cortical regions of interest (ROIs) are defined by Schaefer’s parcellation (Schaefer et al., 2018) and assigned to 17 functional networks (Thomas Yeo et al., 2011). (b) Subcortical ROIs are defined by FreeSurfer (Fischl et al., 2002).

We defined 400 cortical regions of interest (ROIs) (Schaefer et al., 2018) and 19 subcortical ROIs (Fischl et al., 2002) for each subject (Figure 1). RSFC was measured by Pearson’s *r* correlations between the mean time series of each pair of ROIs. We averaged RSFC within and between 17 subnetworks (Thomas Yeo et al., 2011) and aggregated across the 8 canonical networks (yielding 36 network–network RSFC metrics). Specifically, within-network connectivity was assessed by averaging the pairwise RSFC of all regions assigned to that network. ‘Between’ network connectivity was assessed by computing the pair-wise correlations of each ROI in one network (e.g. frontoparietal) to each ROI in the other network (e.g. default) and averaging across them. We also averaged the time series across all voxels within each of the 19 subcortical ROIs, and computed mean connectivity between each cortical network and each subcortical ROI (yielding 152 network–subcortical RSFC metrics). This results in 188 RSFC metrics for each subject. All RSFC values were Fisher *r*-to-*z* transformed before analysis.

### Covariates

The following covariates were dummy coded, converted to factors, and entered into each LME model: participant’s ethnicity (Hispanic: Yes = 1, No = 0) and sex (female = 0, male = 1). The following covariates were continuous and entered into each LME model: the mean parental education (computed as the average of the highest education degrees across both parents), participant’s age at baseline, and mean FD. To parse out differential predictors of each symptom (i.e. anxiety and depression) over time, we ran a separate set of analyses accounting for the time-varying co-occurrence of the other symptom (depression/anxiety) across the three time points when predicting each symptom (anxiety/depression) over time.

### Imputation of missing data

Missing values in the behavioral data were imputed using the *missForest* algorithm (Stekhoven & Bühlmann, 2012), an iterative imputation method based on random forest. Additional imputation diagnostics are provided in Supplemental Method 2.

### Statistical analyses

First, we constructed LME models with random intercepts using the *lme4* package in Rv4.2.0 (Bates, Mächler, Bolker, & Walker, 2015) to assess if lifetime frequency and severity of any stressor characteristics at baseline assessment predicted each symptom (i.e. anxiety and depression) across three time points (baseline, 6-month, and 12-month follow-up assessments). Each LME model considered the five stressor characteristics simultaneously. To parse off the shared variances between anxiety and depression, we reran the same set of analyses by adding time-varying co-occurrence of depression and anxiety symptoms as covariates.

Second, we tested bivariate associations between each RSFC metric (including within-network, between-network, and network-subcortical) and each predictive stressor characteristic using Pearson’s correlations. To control for multiple comparisons in this screening step, we applied false discovery rate (FDR) correction (Benjamini–Hochberg) within each predictive stressor characteristic and retained RSFC metrics surviving FDR for follow-up analyses. We then evaluated if each stressor-linked RSFC metric screened from the previous step is associated with anxiety and depression symptoms across the three assessment waves using LME models.

Continuous predictors and covariates were standardized to make the beta estimates more interpretable. We used the participant ID as the random intercept, meaning that the intercept estimates were allowed to vary across participants while the beta estimate for each predictor was constant across participants.

Since the LME model residuals were slightly non-normal and heteroscedastic (Supplementary Figures 6–9 and Supplementary Results 1), all analyses were repeated after a square-root transformation was applied to the symptom outcomes to achieve normality and homoscedasticity (Supplementary Figures 10–14 and Supplementary Results 1). Results were largely similar between the two sets of analyses (Figures 2–4).

**Figure 2.**
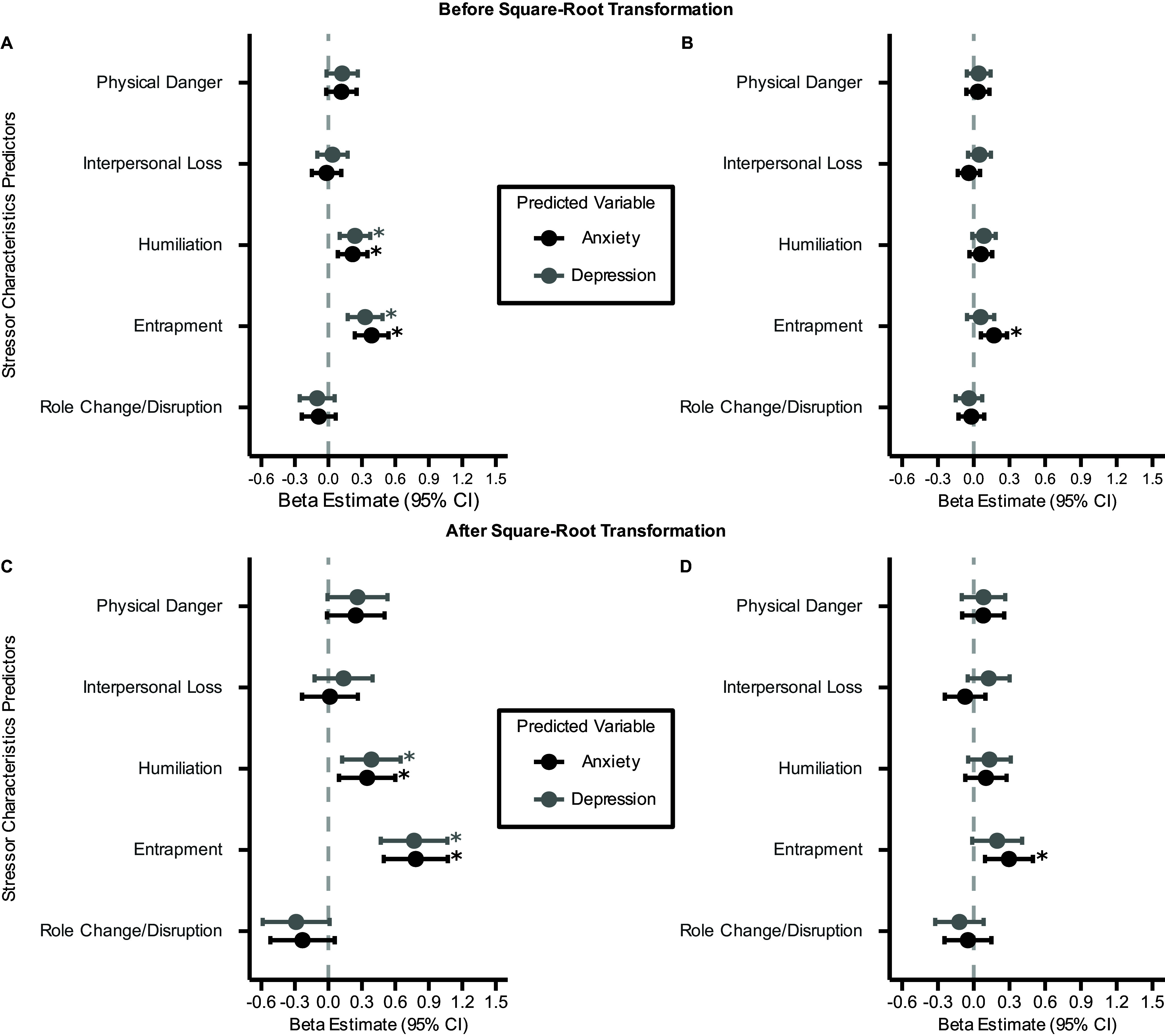
Comparison of standardized *β* estimates (±95% confidence intervals) from LME models predicting anxiety and depression symptoms across three time points from total lifetime frequency of five stressor characteristics at baseline assessment. The vertical dashed line represents *β* = 0. All LME models include a random intercept for each participant and adjust for potentially confounding covariates. Positive *β* estimates indicate that greater stressor frequency is associated with higher symptom scores. Panel (a) and (c) present results without accounting for the co-occurrence of the other symptom, while Panel (b) and (d) present results after accounting for the co-occurrence of the other symptom. *: *q*_FDR_ < 0.05.

**Figure 3.**
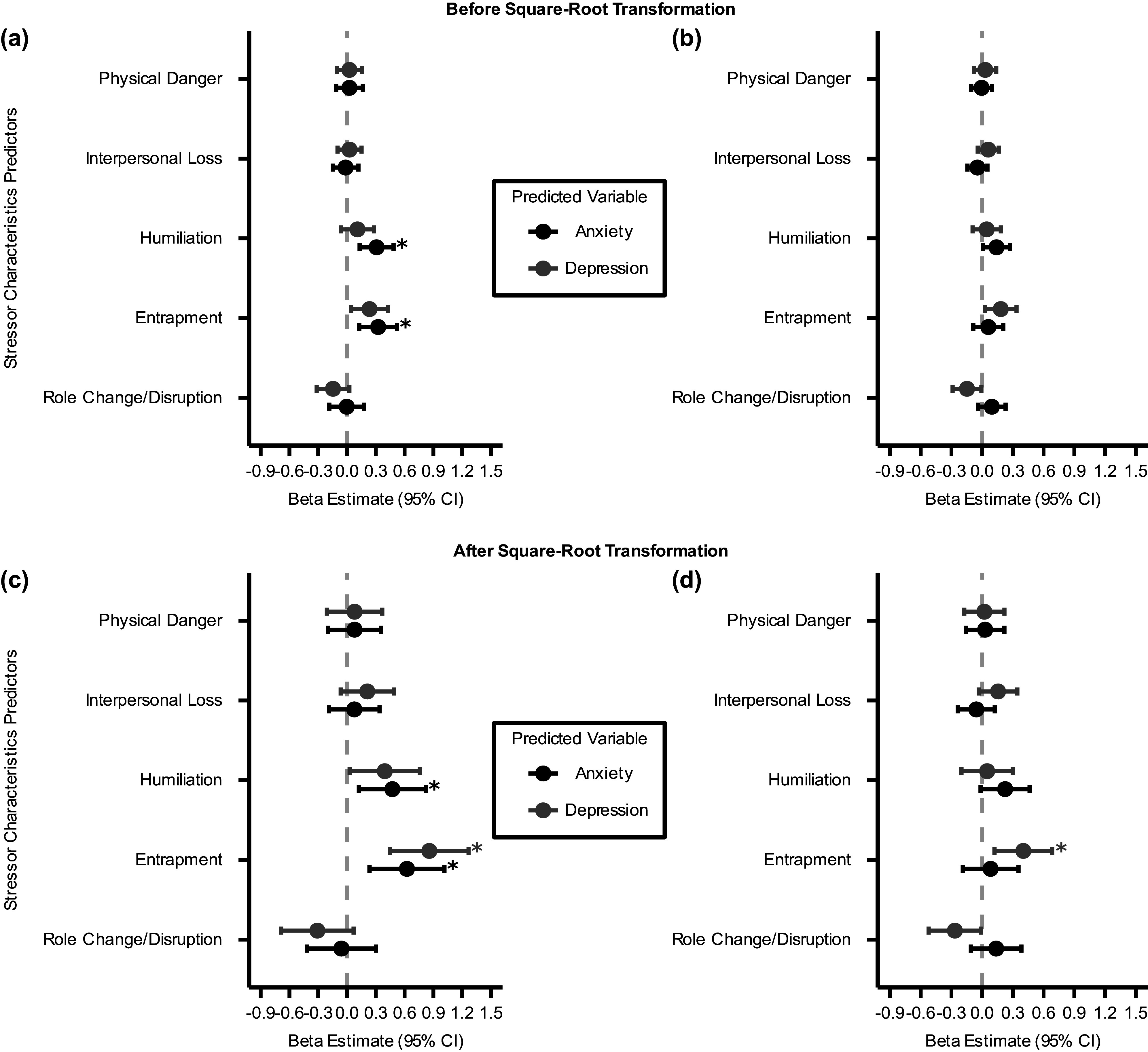
Comparison of standardized *β* estimates (± 95% confidence intervals) from LME models predicting anxiety and depression symptoms at three time points from total lifetime severity of five stressor characteristics at baseline assessment. The vertical dashed line represents *β* = 0. All LME models include a random intercept for each participant and adjust for potentially confounding covariates. Positive *β* estimates indicate that greater stressor severity is associated with higher symptom scores. Panel (a) and (c) present results without accounting for the co-occurrence of the other symptom, while Panel (b) and (d) present results after accounting for the co-occurrence of the other symptom. *: *q*_FDR_ < 0.05.

**Figure 4.**
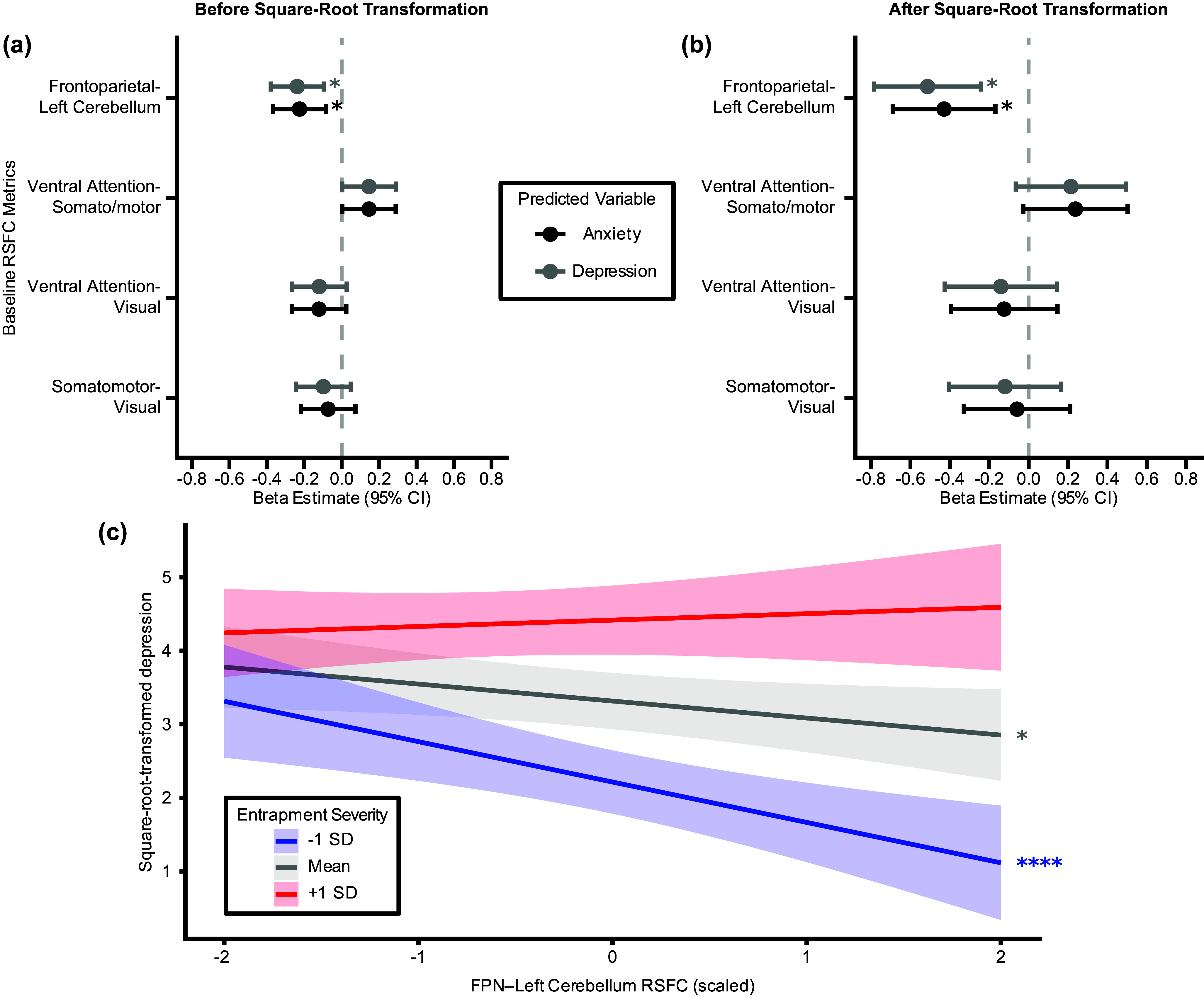
Comparison of standardized *β* estimates (± 95% confidence intervals) from LME models predicting anxiety and depression symptoms at three time points from RSFC at baseline assessment. The four RSFC metrics on the *y*-axis are significantly correlated with entrapment severity: between-network connectivity of the ventral attention (VAN), somatomotor (SOM), and visual (VIS) networks, and RSFC between the frontoparietal network and the left cerebellum (FPN-LeftCerebellum). The vertical dashed line represents *β* = 0. All LME models include a random intercept for each participant and adjust for potentially confounding covariates. Positive *β* estimates indicate that greater RSFC is associated with higher symptom scores. Panel (a) presents results with/without square-root transformation, while Panel (b) demonstrates the association between frontoparietal-left cerebellar RSFC and depression symptoms at different levels of entrapment severity. Panel (c) demonstrates how the association between lifetime entrapment severity and depression symptoms varies as a function of frontoparietal–left cerebellar RSFC. *: *q*_FDR_ < 0.05.

## Results

### Greater total lifetime frequency and severity of humiliation and entrapment exposures predict higher anxiety and depression symptoms across time

We used total lifetime frequency and severity ratings of all stressor social-psychological characteristics (i.e. physical danger, interpersonal loss, humiliation, entrapment, and role change/disruption) at baseline to predict each symptom (i.e. anxiety and depression) across three time points (baseline, 6-month, and 12-month follow-up assessments). After accounting for lifetime frequency of four other stressor characteristics and other potential confounding covariates, greater total lifetime frequency of humiliation and entrapment exposures at baseline assessment prospectively predicted higher anxiety (Humiliation: 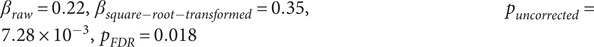 ; Entrapment: 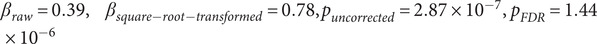 , Figure 2a,c) and depression (Humiliation: 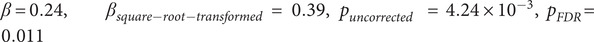 ; Entrapment: 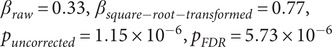 , Figure 2a,c) symptoms.

Results from LME models assessing total lifetime severity of the five stressor characteristics as predictors showed similar patterns. After accounting for lifetime severity of four other stressor characteristics and other potential confounding covariates, greater humiliation and entrapment severity at baseline assessment prospectively predicted higher anxiety symptoms (Humiliation: 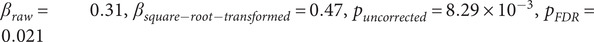 ; Entrapment: 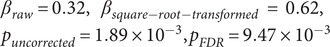 , Figure 3a,c). Although the prospective associations between the lifetime severity of both stressor characteristics and depression symptoms were also positive and significant before correcting for multiple comparisons, only the main effects of entrapment severity survived FDR corrections (Humiliation: 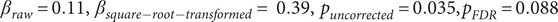 ; Entrapment: 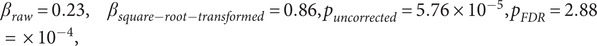 Figure 3a,c).

Overall, these results show that both self-reported frequency and severity of exposures to humiliation and entrapment stressors are uniquely associated with prospective anxiety and depression symptoms over time, controlling for exposures to other stressor characteristics. This suggests that humiliation and entrapment may be the stressor characteristics most strongly related to anxiety and depression in adolescence.

### Total lifetime frequency and severity of entrapment predict anxiety- and depression-specific symptom variances across time

We further tested if the total lifetime frequency of any stressor social-psychological characteristic predicted variances specific to each symptom across three time points by including the time-varying co-occurrence of the other symptom measure as an additional covariate in each LME model. We found that the total lifetime frequency of entrapment stressors predicted anxiety-specific variances across time ( 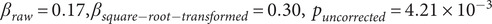 , 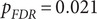 , Figure 2b,d), but not depression-specific variances ( 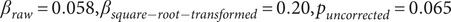 , 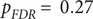 , Figure 2b,d).

We then conducted similar analyses using total lifetime severity of the five stressor characteristics as predictors of anxiety- and depression-specific symptom variances across time. After accounting for time-varying, concurrent depression symptoms, the main effects of humiliation and entrapment severity predicting prospective anxiety symptoms were not significant (Humiliation: 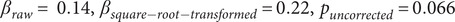 ; Entrapment: 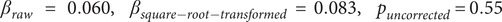 , Figure 3b,d). Greater total lifetime severity of entrapment predicted higher depression symptoms after including concurrent anxiety symptoms as a covariate (Entrapment: 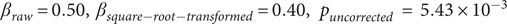 , 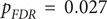 , Figure 3b,d).

These results suggest that exposures to entrapment stressors may be a shared risk factor for both anxiety and depression in adolescence, but whether it is associated with symptom-specific variances depends on the measure used to assess such exposures. On top of the shared variance between anxiety and depression symptoms and controlling for exposures to other stressor characteristics, the number of entrapment exposures is associated with anxiety-specific symptom variances over time, whereas the subjective severity rating of entrapment exposures is associated with depression-specific symptom variances.

### Aberrant frontoparietal-left cerebellar functional connectivity predicts anxiety and depression symptoms over time

Given that lifetime frequency and severity of humiliation and entrapment exposures have been associated with longitudinal anxiety and depression symptoms, we identified network–network and network–subcortical RSFC metrics that are significantly associated with lifetime frequency and severity of humiliation and entrapment exposures. We identified four RSFC metrics, which demonstrated significant bivariate correlations with entrapment severity after FDR: RSFC between ventral attention and visual networks (*r* = -0.30, *p*_uncorrected_ = 0.00016, *p*_FDR_ = 0.021), between visual and somatomotor networks (*r* = -0.28, *p*_uncorrected_ = 0.00041, *p*_FDR_ = 0.021), between ventral attention and somatomotor networks (*r* = 0.28, *p*_uncorrected_ = 0.00044, *p*_FDR_ = 0.021), and between frontoparietal network and left cerebellum (*r* = -0.29, *p*_uncorrected_ = 0.00025, *p*_FDR_ = 0.021).

Next, we assessed whether these RSFC metrics predict anxiety and depression symptoms across the three assessment waves. We evaluated each RSFC metric separately using LME models. After accounting for potential confounders, RSFC between the frontoparietal network and the left cerebellum is negatively associated with both anxiety ( 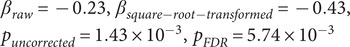 , Figure 4a,b) and depression ( 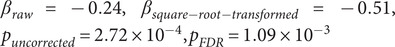 , Figure 4a,b) symptoms across time. There is a positive interaction between lifetime severity of entrapment exposure and RSFC between the frontoparietal network and the left cerebellum ( 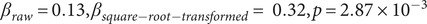 ), such that the negative association between frontoparietal-left cerebellar RSFC and depression symptoms is only significant when entrapment exposure is below average (Figure 4c).

Taken together, more negative frontoparietal–left cerebellar RSFC is linked with greater lifetime severity of entrapment exposures at baseline, and both are also associated with higher anxiety and depression symptoms across time. The RSFC-symptom association is attenuated when lifetime entrapment severity is above average, suggesting that stress exposure moderates the association between frontoparietal-left cerebellar RSFC and adolescent internalizing symptoms.

## Discussion

In this study, we first used multivariable LME models to assess lifetime frequency and severity of exposures to five stressor characteristics at the time of baseline assessment as joint predictors of each symptom (anxiety and depression) across three time points (baseline, 6-month, and 12-month follow-up assessments). Results from these models showed that (a) greater lifetime humiliation and entrapment frequency and severity predicted higher anxiety and depressive symptoms over time; (b) entrapment frequency and severity predicted higher anxiety and depressive symptoms over time after additionally adjusting for concurrent depressive and anxiety symptoms. These findings identify humiliation and entrapment as the stressor characteristics most strongly linked to anxiety and depressive symptoms when considered alongside other stress dimensions, highlighting humiliation- and entrapment-related stress exposures as key risk factors for adolescent internalizing problems. Across all network–network and network–subcortical RSFC metrics, we identified four RSFC metrics significantly coupled with entrapment severity and tested their associations with anxiety and depression symptoms across time. We found that more negative RSFC between the frontoparietal network and the left cerebellum is associated with greater anxiety and depression symptoms over the course of 1 year, and that its association with prospective depression symptoms becomes attenuated with entrapment severity. Our identification of frontoparietal–left cerebellar connectivity as a stress-sensitive marker aligns with growing evidence that the cerebellum plays a key role in cognitive–affective regulation and is implicated in stress-related psychopathology (Schmahmann, Guell, Stoodley, & Halko, 2019). This finding moves beyond a cortico-centric model of psychopathology and points to promising new directions for mechanistic research.

As opposed to prior studies suggesting that exposure to major life stressors characterized by danger is a specific risk factor for anxiety (Asselmann et al., 2015; Ayazi et al., 2014; Finlay-Jones & Brown, 1981; Kendler et al., 2003), we failed to find a significant association between lifetime exposures to physical danger and prospective anxiety symptoms. Moreover, our results did not reveal differential prediction of depressive symptoms by lifetime exposures to loss stressors, as has been previously shown (Asselmann et al., 2015; Farmer & McGuffin, 2003; Finlay-Jones & Brown, 1981; Keller et al., 2007; Kendler et al., 2003). Unlike prior studies that conducted bivariate analyses between specific stressor characteristics and mental health outcomes, our study examined the *unique contributions* of each stressor characteristic to the prediction of anxiety and depression symptoms after accounting for all other stressor characteristics. Taken together, these results suggest that the differential association between physical danger/interpersonal loss and prospective anxiety/depression symptoms may be fully explained by the main effects of exposures to other stressor characteristics.

Our results are consistent with prior studies implying that exposures to humiliation and entrapment may contribute to both anxiety and depression (Griffiths et al., 2014; Kendler et al., 2003; Li et al., 2024; Taylor, Gooding, Wood, & Tarrier, 2011). Our findings go further to show that humiliation and entrapment exposures are the strongest correlates of anxiety and depression symptoms over 1 year when compared to other stressor characteristics. Humiliation is theorized as perceived devaluation of the self, arising from being unfairly harmed by others (Klein, 1991; D. A. Lee, Scragg, & Turner, 2001). Exposures to humiliation may heighten anxiety and depression by increasing vigilance toward potential future harm by others and viewing the self as worthless. Common examples of humiliation stressors include public shaming, peer bullying, social exclusion, rejection, and revelation of infidelity. On the other hand, entrapment refers to the feelings of being chronically trapped in a stressful situation despite having a strong desire to escape (Gilbert & Allan, 1998). Exposures to entrapment stressors may provoke anxiety and depression by fostering a sense of diminished control and reinforcing perceptions of futility, consistent with the concept of learned helplessness. Examples of entrapment stressors include persistent food and financial insecurity, unsafe housing, and overwhelming demands at work or school. Results from the present study highlight the potentially long-lasting impacts of humiliating and socio-structural stressors on both anxiety and depression over time. Our findings suggest that not all stress exposure carries equal risk: Experiences involving social devaluation and ‘no-escape’ contexts may be especially relevant to adolescent internalizing symptoms over time. Moreover, different measures of entrapment exposures showed specific associations with different symptom outcomes, after accounting for their shared variance. This suggests that more frequent entrapment exposures, irrespective of severity, may be more anxio-genic by encouraging hypervigilance, sustaining anticipation of further threat. On the other hand, isolated occurrences of severe entrapment stressors can exhaust one’s coping resources and perpetuate the belief that any escape is futile. Together, these results highlight the potential value of focusing on assessing humiliation- and entrapment-related stress exposures when identifying adolescents at risk for worsening internalizing symptoms.

Lastly, we found that RSFC between the frontoparietal network and the left cerebellum is not only associated with lifetime severity of entrapment exposures at baseline assessment, but also negatively associated with anxiety and depression symptoms over the course of 1 year. A few studies have similarly revealed negative associations between prefrontal-left cerebellar RSFC and internalizing psychopathology (Luo et al., 2018; MinlanYuan et al., 2017; Wang et al., 2023). Our findings extend this literature by suggesting that the relationship between disruptions in the cerebro-cerebellar circuit and adolescent internalizing symptoms may be linked to chronic exposure to stressors that are inescapable. The cerebellum demonstrates strong intrinsic coupling with association networks, including frontoparietal and default networks. Accordingly, frontoparietal–left cerebellar RSFC can be interpreted as a network-level index of cerebro-cerebellar coupling within canonical association systems (Buckner et al., 2011).

Importantly, clinic-based evidence suggests that cerebellar dysfunction can disrupt modulatory processes that calibrate cognition and emotion, producing affective and behavioral phenotypes that overlap with internalizing psychopathology (Schmahmann, Weilburg, & Sherman, 2007). Our findings broaden the cortico-centric focus in the existing literature and suggest that exposures to inescapable, persistent stressors may strain cerebellar modulatory control, which can have implications for adolescent internalizing problems over time. However, our findings should be interpreted with caution, because lifetime stress exposure and RSFC were assessed concurrently at baseline in our study. These findings should be interpreted as identifying stress-linked connectivity correlates rather than establishing a prospective mediation or causal pathway, motivating the need for future multiwave neuroimaging studies.

### Strengths and limitations

Several strengths and limitations of this work should be noted. In terms of strengths, we leveraged a longitudinal sample of adolescents enriched for clinical diagnosis of anxiety and depressive disorders with a narrow age range. This enabled us to examine prospective associations between exposure to major life stressors, neurobiology, and clinical symptoms during the developmental stage characterized by heightened vulnerability to stress-related psychopathology (Casey et al., 2008; Larsen & Luna, 2018; March-Llanes et al., 2017; Merikangas et al., 2010; Paus et al., 2008). We assessed a rich repertoire of theoretically relevant stressors occurring across the entire life course using distinct dimensions of social-psychological characteristics assessed by the STRAIN. Our study had a novel focus on using subjective severity ratings, in addition to the number of exposures to stressor characteristics. In this way, we demonstrated the validity of these stressor characteristic indices in predicting internalizing psychopathology over time. We included all five social-psychological characteristics and the potential confounding variables into the same LME model when predicting anxiety/depression symptoms at three time points. This ensured that any prospective association between a stressor characteristic and clinical symptom is robust to the inclusion of other intercorrelated stressor characteristics. Lastly, each participant in our study underwent 23.2 minutes of rsfMRI. The scan duration of our rsfMRI data goes far beyond that of most previous rsfMRI studies and should afford relatively high reliability (Birn et al., 2013).

Several limitations should also be noted. First, the sample used for brain-symptom analyses was relatively small (*n* = 130), which may be underpowered for detecting brain-behavior relationships that are reproducible and generalizable (Marek et al., 2022). Although a small sample size is a common issue with richly phenotyped, longitudinal datasets involving clinical samples, findings from the present study should be validated by larger samples in future studies. Since the fMRI protocol of the current dataset was harmonized with other HCP studies (Siless et al., 2020), other HCP datasets may provide valuable resources for this purpose. Second, the vast majority (79.33%) of participants were White, came from highly educated families, and endorsed relatively low exposures to stressors characterized by danger and role change/disruption (Table 1) compared to previous studies on autistic adults (Moseley et al., 2025) and sexual minority adults of color (Parra et al., 2023). Although our study assessed moderate-to-severe life stressors, and previous research suggests that exposure to a single severe stressor can trigger depressive episodes in over half of individuals (Monroe, Slavich, Torres, & Gotlib, 2007; Shapiro, 1979), further research is still needed to confirm whether our findings generalize to populations with greater exposure to stressors involving danger and significant role disruptions. Third, the lifetime frequency and severity of stress exposures were assessed retrospectively. This may have introduced recall bias. Lastly, although we included a number of covariates such as age, race, ethnicity, baseline symptoms, and diagnostic status in our LME models to ensure that our findings could not be explained by these confounders, other potential confounders, such as medication use and family history of psychopathology, were unavailable in this dataset and may be relevant.

### Conclusion

Notwithstanding these limitations, through the use of a longitudinal sample of adolescents, over half of whom met clinical cutoffs for at least one anxiety or depressive disorder, the present study highlights entrapment – and to a lesser extent humiliation – as stressor characteristics that show the most robust prospective associations with adolescent internalizing symptoms over the course of one year when modeled alongside other stressor domains. These results show that chronic exposures to stressors involving social devaluation and ‘no-escape’ contexts may be especially relevant to adolescent internalizing symptoms over time. Follow-up analyses identified frontoparietal–left cerebellar coupling as a connectivity feature linked to entrapment severity and to subsequent anxiety and depressive symptoms over time, with evidence that the RSFC–symptom association varies as a function of entrapment severity. These findings underscore the value of moving beyond cumulative stress exposures to characterize stressor dimensions that may be most clinically salient, and they implicate cerebro-cerebellar control circuitry as a stress-sensitive RSFC marker of adolescent internalizing problems.

## Supporting information

10.1017/S0033291726103699.sm001 Qu et al. supplementary material Qu et al. supplementary material

## Supplementary Material

The supplementary material for this article can be found at http://doi.org/10.1017/S0033291726103699.
