# Supplementary material for: Life experiences of humiliation, entrapment, and frontoparietal-cerebellar connectivity predict adolescent anxiety and depression symptoms
Source: Psychol Med. 2026 Mar 30;56:e84. doi: 10.1017/S0033291726103699 (PMC13079225; doi:10.1017/S0033291726103699)

Supplementary Materials for

**Lifetime entrapment and humiliation exposures and frontoparietal-cerebellar coupling predict adolescent anxiety and depression symptoms**

Yueyue Lydia Qu^1,2^, Sidhant Chopra^3,4^, Shijie Qu^1,2^, Carrisa V. Cocuzza^5^, Loïc Labache^5^, Clemens C.C. Bauer ^6,7,8^, Francesca Morfini^9,10^, Susan Whitfield-Gabrieli^6,7,8^, George M. Slavich^11^, Jutta Joormann^1,2^, & Avram J. Holmes^5^

^1^ Department of Psychology, Yale University, New Haven, CT, USA

^2^ Wu Tsai Institute, Yale University, New Haven, CT, USA

^3^ Orygen, Melbourne, VIC, Australia

^4^ Centre for Youth Mental Health, The University of Melbourne, Melbourne, VIC, Australia

^5^ Department of Psychiatry, Brain Health Institute, Rutgers University, Piscataway, NJ, USA

^6^ Department of Psychology, Northeastern University, Boston, MA, USA

^7^ Center for Cognitive & Brain Health, Northeastern University, Boston, MA, USA

^8^ Department of Brain and Cognitive Sciences and McGovern Institute for Brain Research, Massachusetts Institute of Technology, Cambridge, MA, USA

^9^ Center for Depression, Anxiety and Stress Research, McLean Hospital, Belmont, MA, USA

^10^ Department of Psychiatry, Harvard Medical school, Boston, MA, USA

^11^ Department of Psychiatry and Biobehavioral Sciences, University of California, Los Angeles, CA, USA

**Supplementary Method 1. Brief definitions of the five stressor characteristics from adolescent STRAIN**

**Physical danger**. The degree of potential future threat to one’s physical safety that might occur as a result of the stressor.

**Interpersonal loss**. Diminution of a sense of connectedness or well-being as a result of a real or realistically imagined loss of a person by death or by separation.

**Humiliation**. The likelihood of a stressor rendering a person devalued in relation to others or self, usually due to rejection or a sense of core failure.

**Entrapment**. Ongoing circumstances of marked difficulty of at least 6 months’ duration that the individual can reasonably expect to persist or get worse, with little or no possibility that a resolution can be achieved as a result of anything that might reasonably be done.

**Role change/disruption**. Life transitions that involve addition, subtraction or change of social roles.

**Supplementary Method 2. Imputation of missing behavioral data using missForest algorithm**

The *missForest* algorithm was selected for its ability to impute both numeric and factor data and for its superior performance compared to 15 other common imputation algorithms as evaluated by the *missCompare* framework (Varga, 2020). Under the *missCompare* framework, 50 simulated datasets matching multivariate characteristics and missingness patterns of the original data were generated. The missing data in each simulated dataset were then imputed using a curated list of 16 algorithms. The computation time and imputation accuracy of each algorithm were hitherto assessed by calculating Root Mean Square Error (RMSE), Mean Absolute Error (MAE) and Kolmogorov-Smirnov (KS) values between the imputed and the simulated data points (**Supplementary Figure 4**). These metrics were compared under three conditions: Missing Completely At Random (MCAR), Missing At Random (MAR), and Missing Not At Random or Non-Ignorable (MNAR). Finally, post-imputation diagnostics, including visual examination of data distributions and stability of correlation coefficients between measures, before and after imputation, were examined, showing minimal impact of imputation (**Supplementary Figure 5**).

**Supplementary Result 1. Test of normality of LME model residuals before and after square-root transformation**

Besides visually examining the normal Q-Q plots using LME model outputs to assess normality of model residuals, we also conducted two-sample Kolmogorov-Smirnov tests to statistically compare the LME model residuals to the normal distribution. Before square-root transformation, two-sample Kolmogorov-Smirnov tests showed that residuals of the LME models predicting anxiety (*D*s = 0.085, 0.086, 0.084, 0.088, 0.088, 0.087; *p*s = 0.0031, 0.0026, 0.0086, 0.0047, 0.0045, 0.0055) and depression (*D*s = 0.088, 0.094, 0.098, 0.095, 0.099, 0.094; *p*s = 0.0020, 0.00074, 0.0010, 0.0018, 0.0010, 0.0020) symptoms did not come from the normal distribution. After square-root transformation, two-sample Kolmogorov-Smirnov tests showed that residuals of the LME models predicting anxiety (*D*s = 0.053, 0.050, 0.054, 0.049, 0.052, 0.052; *p*s = 0.16, 0.21, 0.20, 0.30, 0.23, 0.24) and depression (*D*s = 0.034, 0.032, 0.031, 0.049, 0.044, 0.046; *p*s = 0.68, 0.75, 0.85, 0.30, 0.44, 0.39) symptoms were not significantly different from the normal distribution.

**Supplementary Table 1**. Bivariate correlations between continuous variables in the LME models

|  | **Interpersonal loss severity** | **Humiliation severity** | **Entrapment**  **severity** | **Role reversal severity** | **bFPN-LC FC** | **bVAN-SOM FC** | **bVAN-VIS FC** | **bSOM-VIS FC** | **Anxiety at baseline** | **Anxiety at time 1^a^** | **Anxiety at time 2^b^** | **Depression at baseline** | **Depression at time 1 ^a^** | **Depression at time 2 ^b^** | **Mean parental education** | **Baseline Age** |
| --- | --- | --- | --- | --- | --- | --- | --- | --- | --- | --- | --- | --- | --- | --- | --- | --- |
| **Physical danger severity** | **0.52****** | **0.67****** | **0.6****** | **0.63****** | -0.14 | **0.21*** | -0.12 | -0.13 | **0.55****** | **0.43****** | **0.36****** | **0.51****** | **0.44****** | **0.33****** | **-0.17*** | 0.13 |
| **Interpersonal loss severity** |  | **0.58****** | **0.6****** | **0.67****** | **-0.26***** | **0.17*** | **-0.17*** | -0.15 | **0.52****** | **0.4****** | **0.32****** | **0.48****** | **0.42****** | **0.33****** | -0.13 | 0.16 |
| **Humiliation severity** |  |  | **0.78****** | **0.67****** | **-0.26***** | **0.2*** | -0.15 | **-0.17*** | **0.71****** | **0.59****** | **0.48****** | **0.65****** | **0.56****** | **0.49****** | -**0.21**** | 0.06 |
| **Entrapment severity** |  |  |  | **0.8****** | **-0.29****** | **0.28****** | **-0.3****** | **-0.28****** | **0.73****** | **0.61****** | **0.55****** | **0.67****** | **0.59****** | **0.56****** | -0.13 | **0.21*** |
| **Role reversal severity** |  |  |  |  | **-0.19*** | **0.23***** | **-0.24***** | **-0.27****** | **0.62****** | **0.52****** | **0.44****** | **0.53****** | **0.47****** | **0.42****** | **-0.16*** | **0.22**** |
| **bFPN-LC FC** |  |  |  |  |  | **-0.17*** | 0.14 | **0.21*** | **-0.23***** | **-0.20*** | **-0.22*** | **-0.20*** | **-0.18*** | **-0.26***** | 0.09 | -0.01 |
| **bVAN-SOM FC** |  |  |  |  |  |  | **-0.36****** | **-0.3****** | **0.23***** | **0.16*** | 0.11 | **0.19*** | 0.16 | 0.15 | 0.02 | 0.03 |
| **bVAN-VIS FC** |  |  |  |  |  |  |  | **0.51****** | **-0.18*** | -0.14 | -0.08 | **-0.19*** | -0.13 | -0.13 | 0.16 | -0.05 |
| **bSOM-VIS FC** |  |  |  |  |  |  |  |  | **-0.17*** | -0.07 | -0.02 | **-0.17*** | -0.04 | -0.1 | 0.07 | 0.01 |
| **Anxiety at baseline** |  |  |  |  |  |  |  |  |  | **0.73****** | **0.64****** | **0.79****** | **0.65****** | **0.61****** | -0.1 | **0.18*** |
| **Anxiety at time 1^a^** |  |  |  |  |  |  |  |  |  |  | **0.8****** | **0.58****** | **0.83****** | **0.69****** | -0.03 | **0.24***** |
| **Anxiety at time 2^b^** |  |  |  |  |  |  |  |  |  |  |  | **0.58****** | **0.7****** | **0.85****** | 0.07 | **0.22**** |
| **Depression at baseline** |  |  |  |  |  |  |  |  |  |  |  |  | **0.69****** | **0.69****** | -0.1 | 0.15 |
| **Depression at time 1^a^** |  |  |  |  |  |  |  |  |  |  |  |  |  | **0.75****** | -0.02 | **0.32****** |
| **Depression at time 2^b^** |  |  |  |  |  |  |  |  |  |  |  |  |  |  | 0 | **0.21*** |
| **Mean parental education** |  |  |  |  |  |  |  |  |  |  |  |  |  |  |  | -0.03 |

Note: All statistics are Pearson's correlation coefficient r. **p*<.05 ***p*<0.01****p*<0.005 *****p*<0.001 a. Time 1 = at 6-month follow-up assessment. b. Time 2 = at 12-month follow-up assessment. FC = functional connectivity. bFPN-LC = between frontoparietal network and left cerebellum, bVAN-SOM = between ventral attention and somatomotor networks. bVAN-VIS = between ventral attention and visual networks. bSOM-VIS = between somatomotor and visual networks.

**Supplemental Table 2**. Demographic information for included and excluded participants from BANDA dataset release 1.1

|  | Included | Excluded | *T*- or χ^2^-Statistic | *p*-value |
| --- | --- | --- | --- | --- |
| *N* | 150 | 65 | - | - |
| Age, mean ± SD | 15.44 ± 0.85 | 15.45 ± 0.84 | -0.062 | 0.951 |
| Sex, (% female) | 92 (61.33) | 49 (75.38) | 3.37 | 0.067 |
| Diagnostic group (%) | | | | |
| Anxiety | 58 (38.67) | 27 (41.54) | 8.19 | 0.017 |
| Control | 52 (34.67) | 11 (16.92) |  |  |
| Depression | 40 (26.67) | 27 (41.54) |  |  |
| Race/ethnicity (%) | | | | |
| White | 121 (80.67) | 49 (75.38) | 6.63 | 0.25 |
| Hispanic | 11 (7.33) | 5 (7.69) | 0.00 | 1.00 |

Note: Anxiety = having a current diagnosis of at least one anxiety disorder and no depressive disorder based on DSM-5. Depression = having a current diagnosis of at least one depressive disorder based on DSM-5.

**Supplementary Figure 1.** Histogram of lifetime frequency of each stressor characteristic across the whole sample (N=150)

**
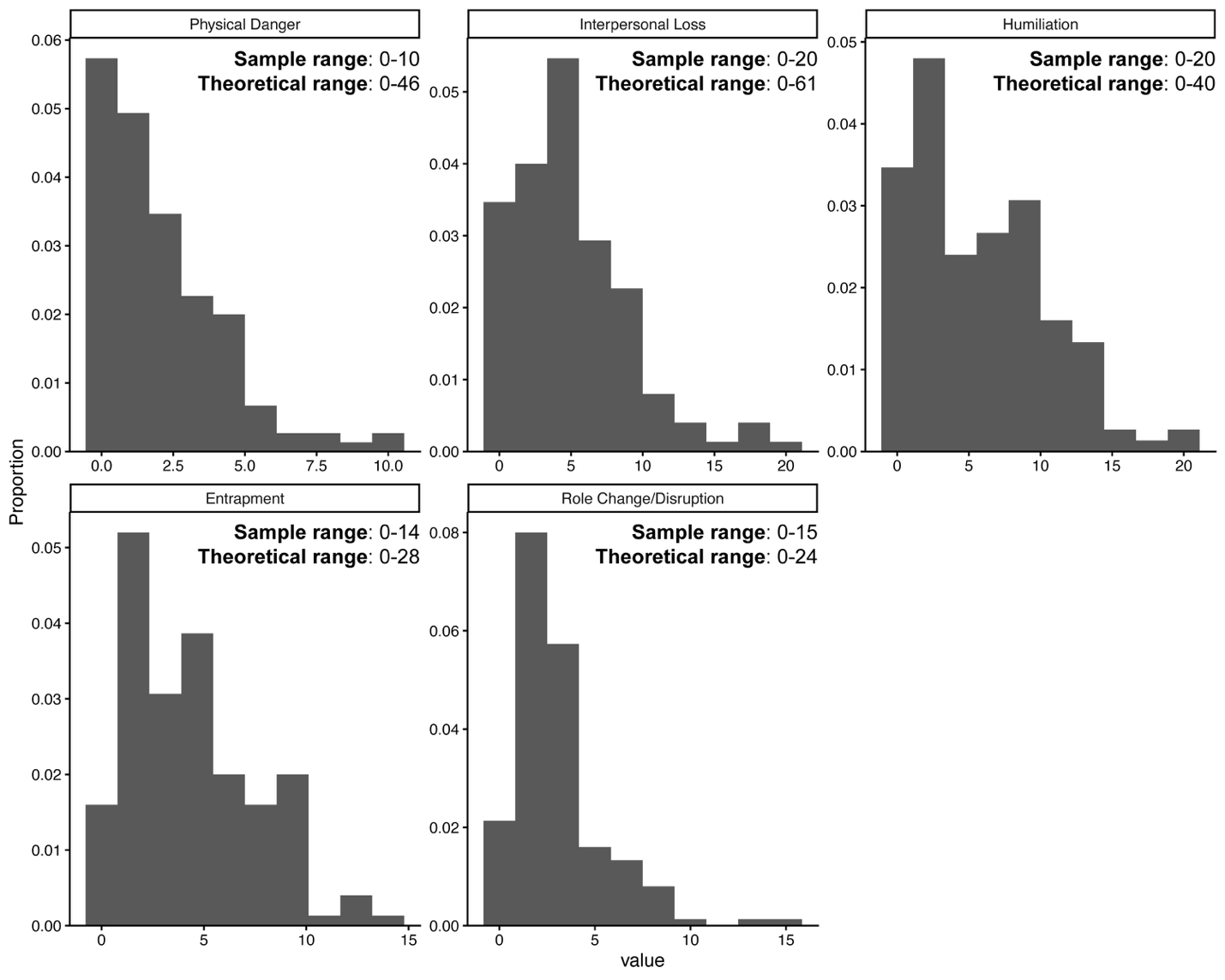
**

**Supplementary Figure 2.** Histogram of lifetime severity of each stressor characteristic across the whole sample (N=150)

**
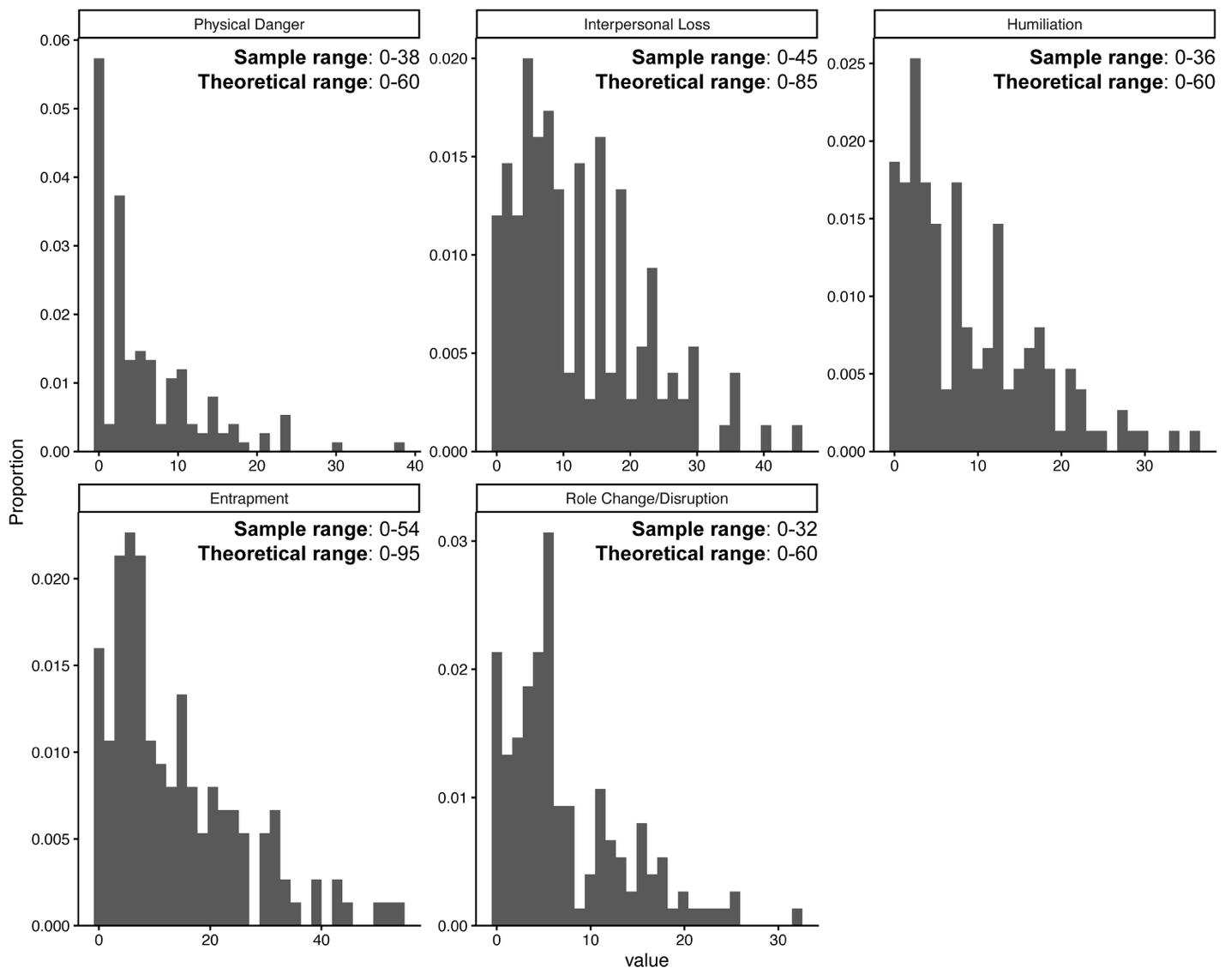
**

**Supplementary Figure 3**. Mean anxiety and depressive symptoms within each diagnostic group at baseline, 6-month follow-up assessment and 12-month follow-up assessment. Anxiety = having a current diagnosis of at least one anxiety disorder and no depressive disorder; Depression = having a current diagnosis of at least one depressive disorder; Control = having no current or lifetime diagnosis of any psychiatric disorder. Shades around each line represent standard error.


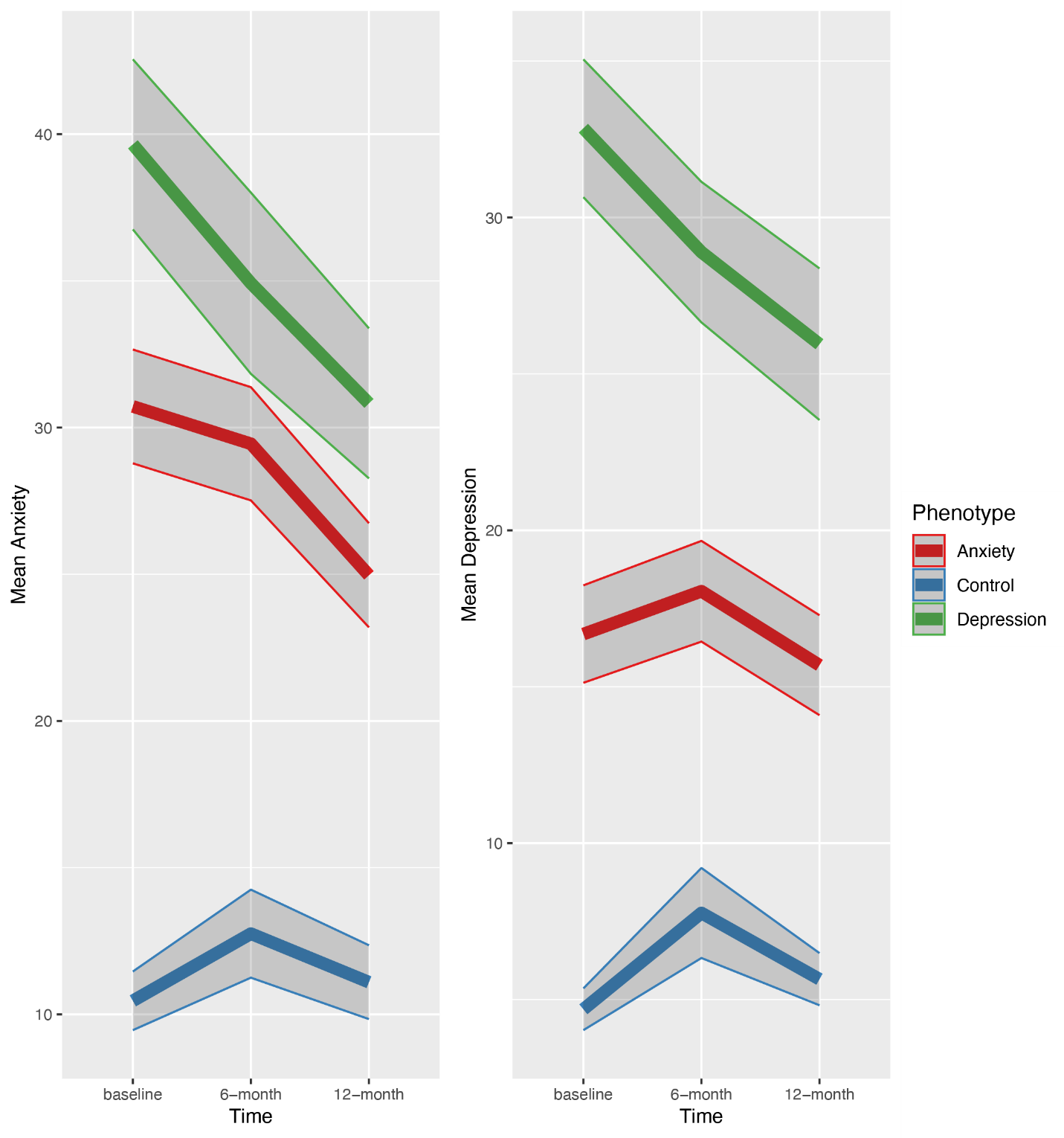


Depression symptoms was computed by the total Mood and Feelings Questionnaire (MFQ), with scores ranging from 0 to 66. Anxiety symptoms were computed by summing the four anxiety subscales (Separation Anxiety Disorder, Social Phobia, Generalized Anxiety and Panic Disorder) from the Revised Children’s Anxiety and Depression Scale (RCADS), ranging from 0 to 93.

**Supplementary Figure 4**. Imputation accuracy (root-mean-square error, mean absolute error, Kolmogorov-Smirnov test statistic) computed by comparing original and imputed data.

Note: Lower values of root-mean-square error, mean absolute error, Kolmogorov-Smirnov test statistic indicate better fit between the original data and imputed data. Some methods (mean imputation, median imputation, missMDA, pcaMethods methods and AmeliaII) do not allow for imputing missing factor variables and therefore inapplicable to our dataset.


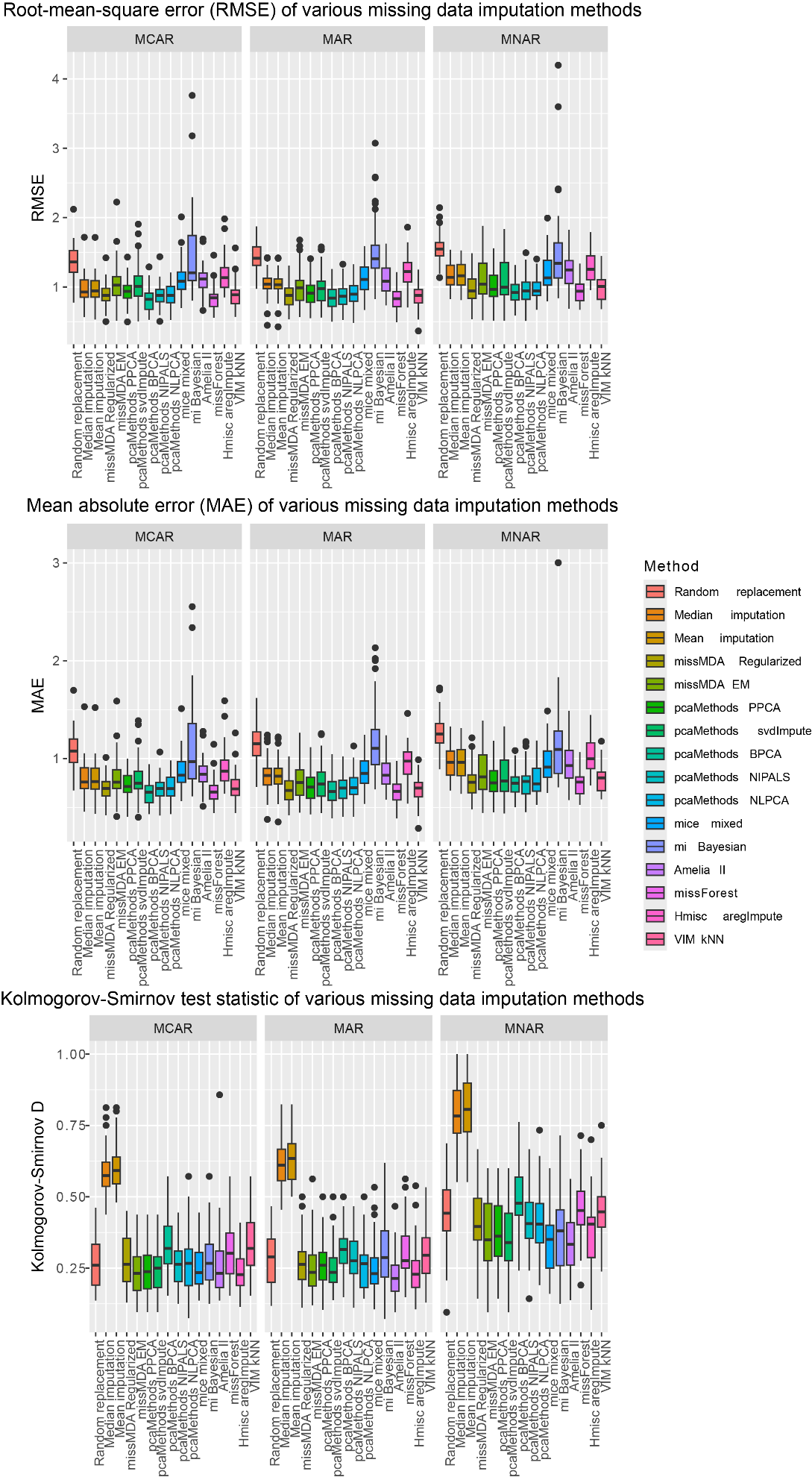


**Supplementary Figure 5**. Bootstrapped correlation coefficients (in red) between the original data and the imputed data. The blue line is a reference line with intercept 0 and slope 1.


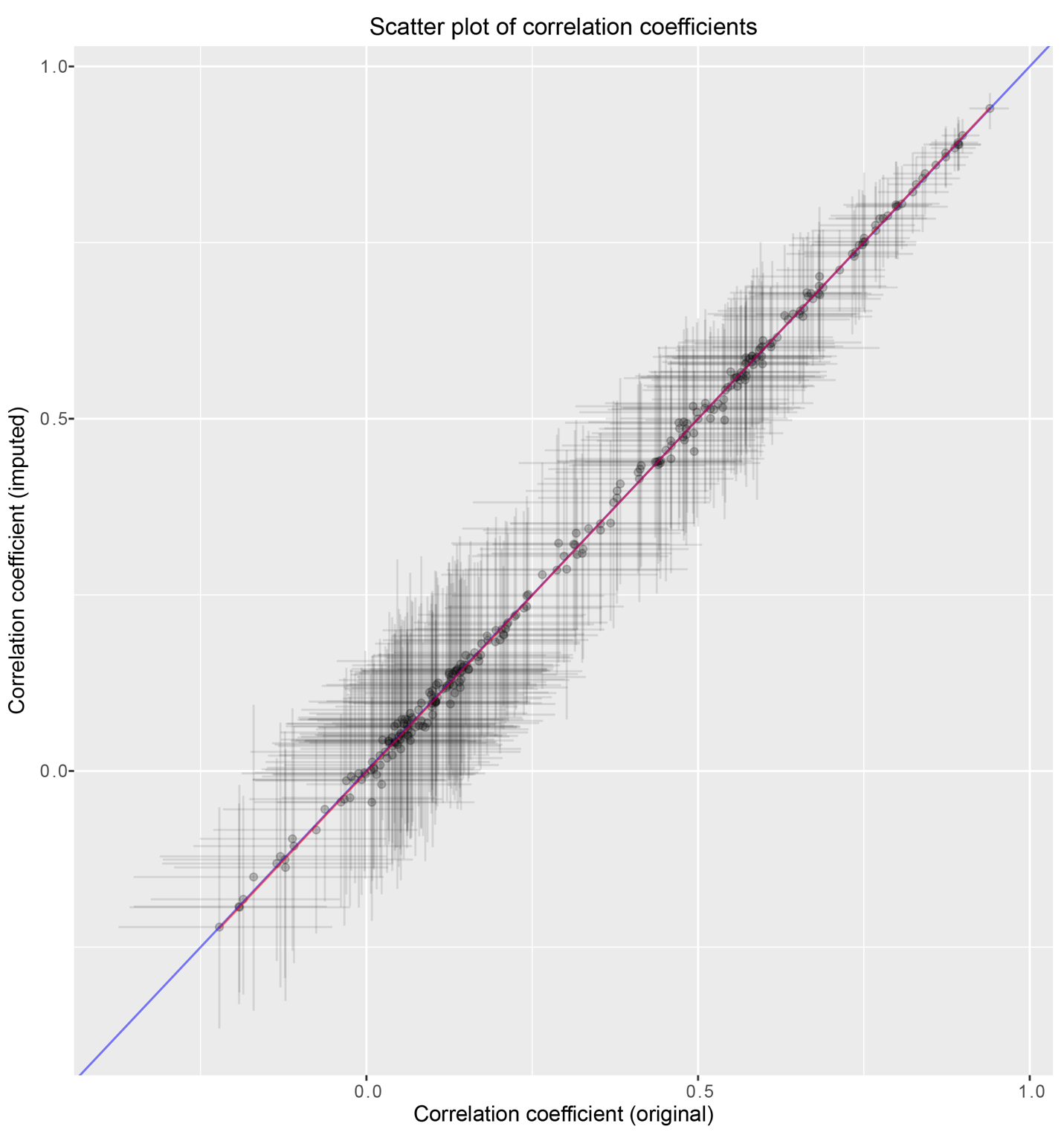


**Supplementary Figure 6**. Q-Q plots and heteroscedasticity plots for LME models predicting anxiety symptoms across three time points from total lifetime frequency (A, C) and severity (B, D) of five stressor characteristics at baseline assessment, **before** square-root transformation


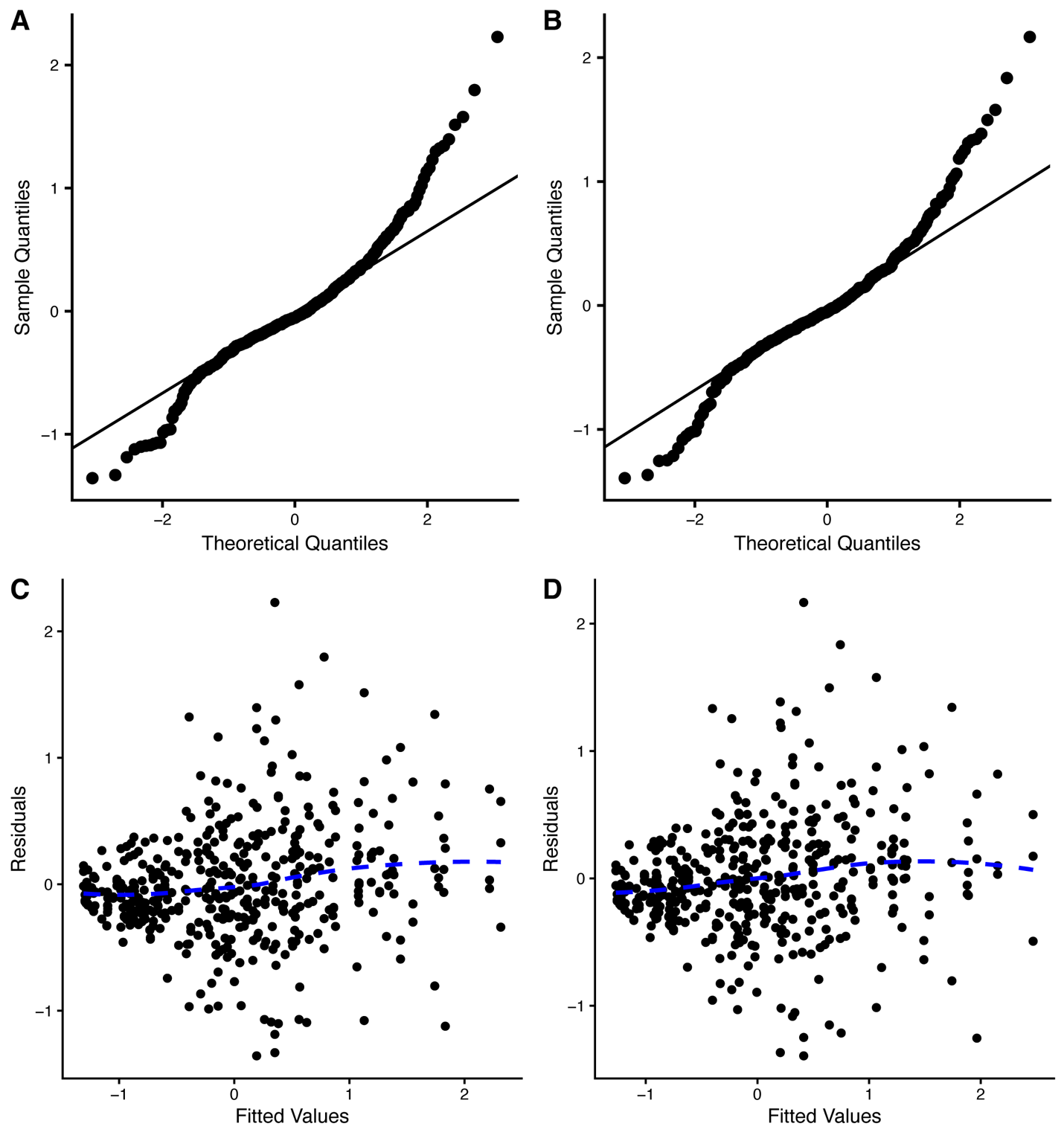


**Supplementary Figure 7.** Q-Q plots and heteroscedasticity plots for LME models predicting anxiety symptoms across three time points from the four RSFC metrics linked to lifetime entrapment severity at baseline assessment, **before** square-root transformation


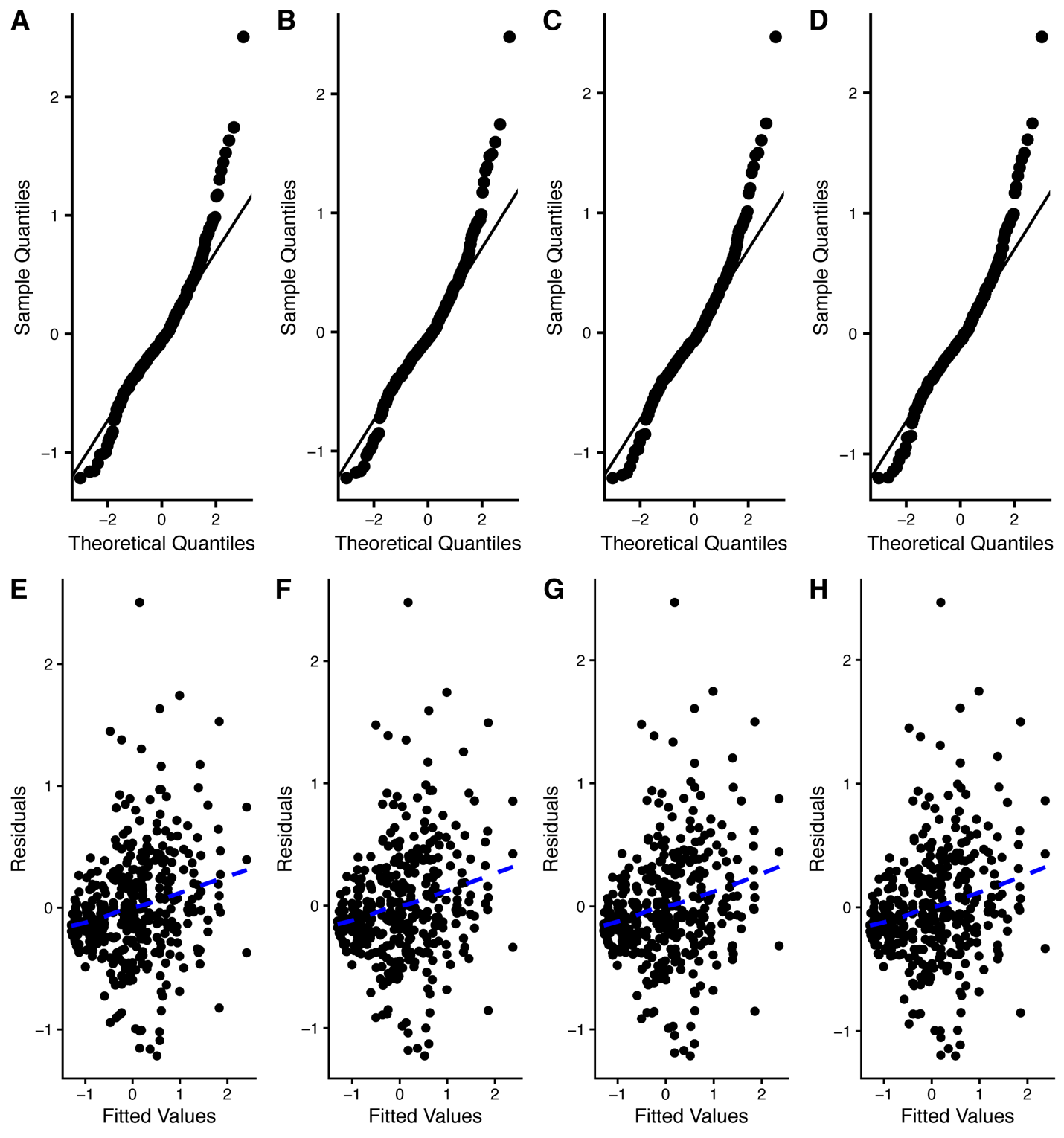


**Supplementary Figure 8**. Q-Q plots and heteroscedasticity plots for LME models predicting depression symptoms across three time points from total lifetime frequency (A, C) and severity (B, D) of five stressor characteristics at baseline assessment, **before** square-root transformation


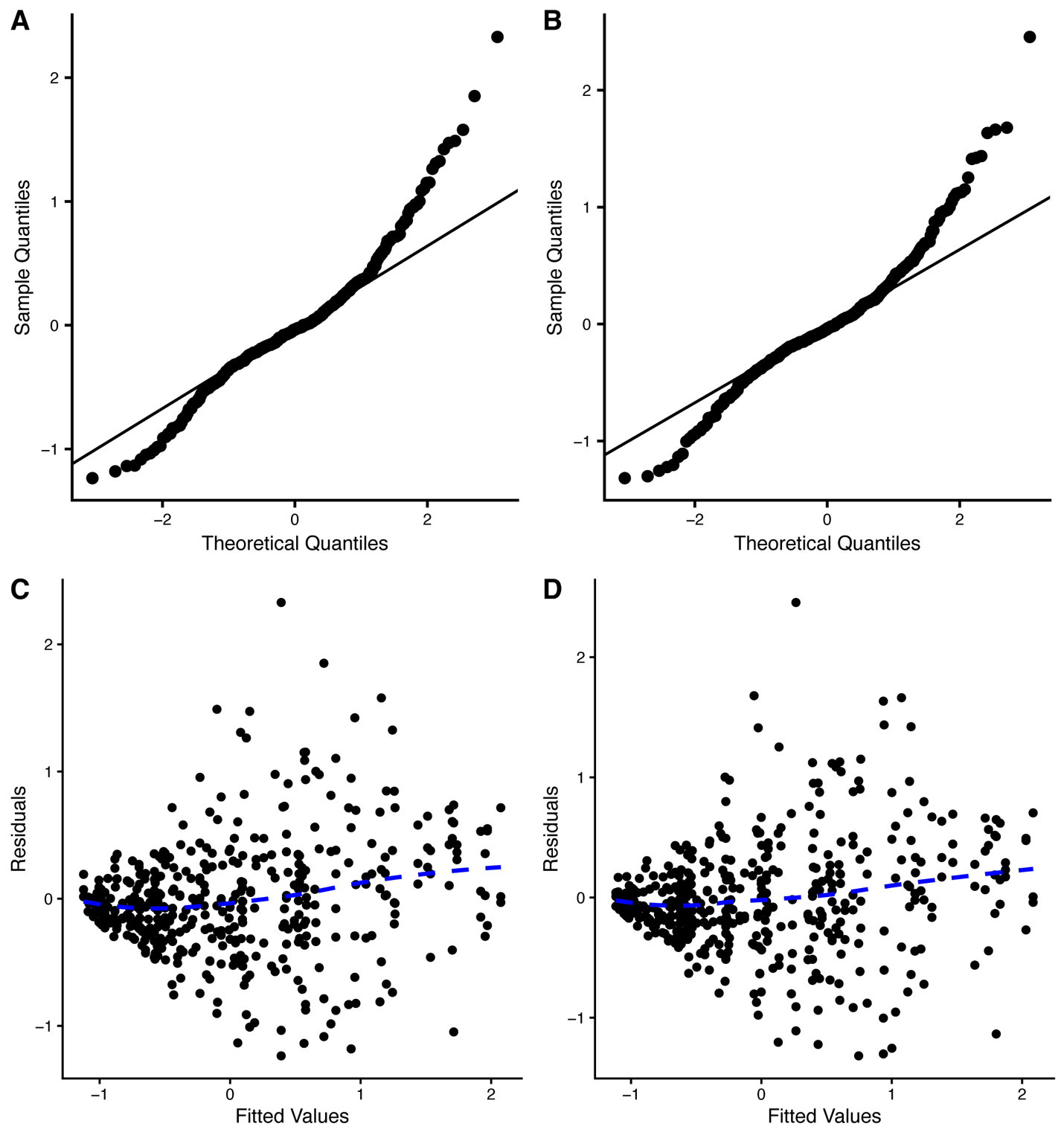


**Supplementary Figure 9**. Q-Q plots and heteroscedasticity plots for LME models predicting depression symptoms across three time points from the four RSFC metrics linked to lifetime entrapment severity at baseline assessment, **before** square-root transformation


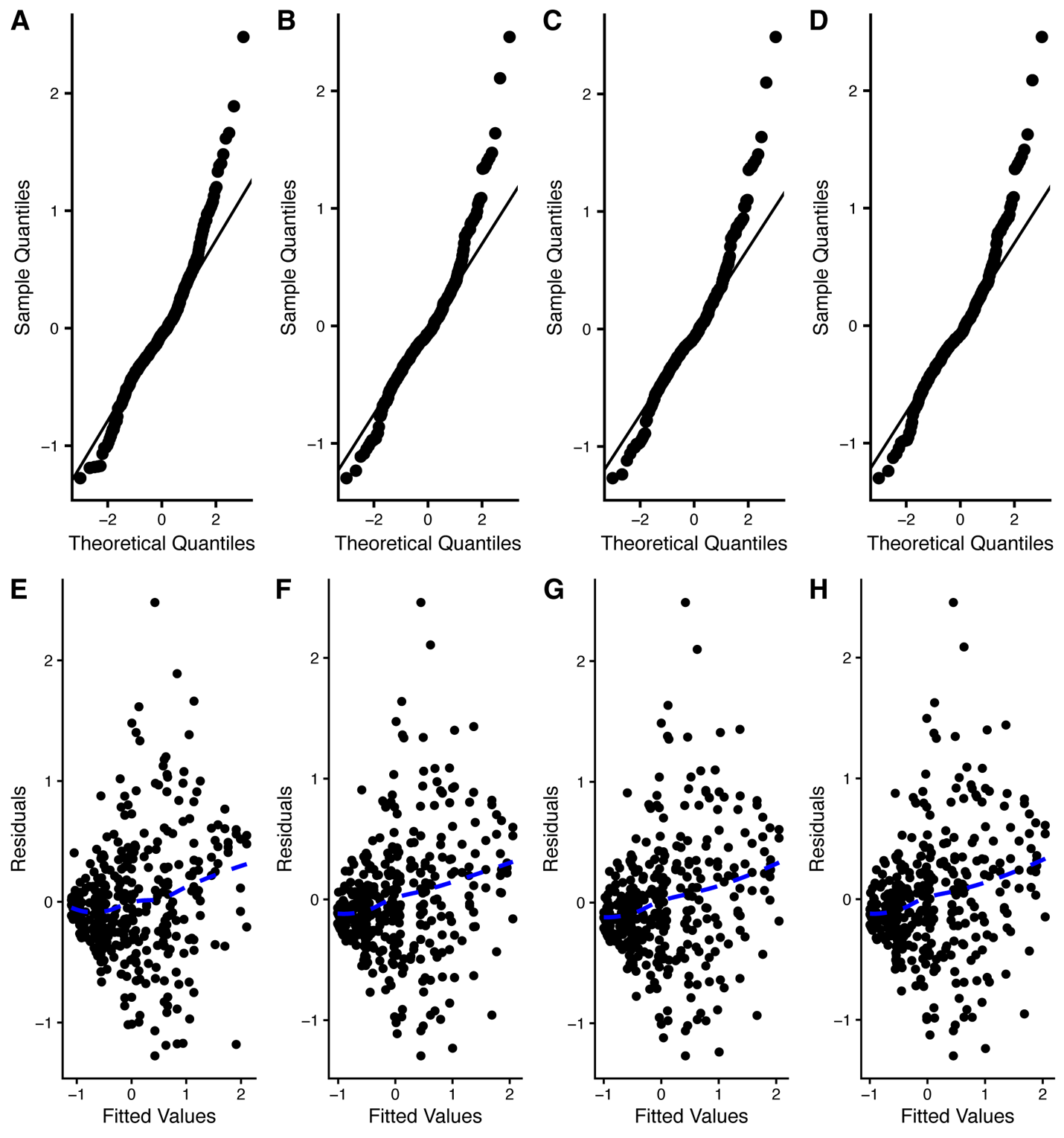


**Supplementary Figure 10**. Q-Q plots and heteroscedasticity plots for LME models predicting anxiety symptoms across three time points from total lifetime frequency (A, C) and severity (B, D) of five stressor characteristics at baseline assessment, **after** square-root transformation


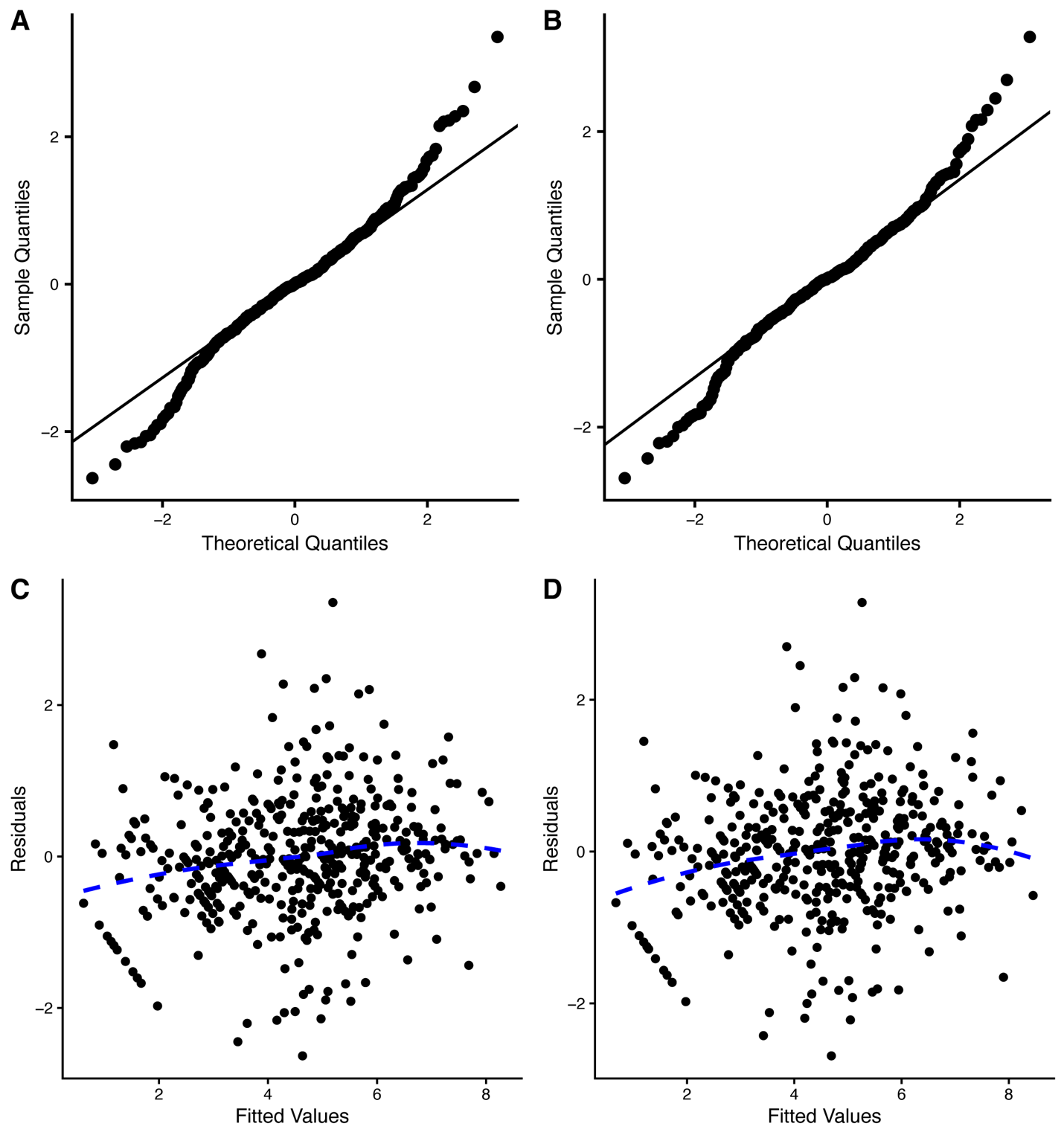


**Supplementary Figure 11.** Q-Q plots and heteroscedasticity plots for LME models predicting anxiety symptoms across three time points from the four RSFC metrics linked to lifetime entrapment severity at baseline assessment, **after** square-root transformation


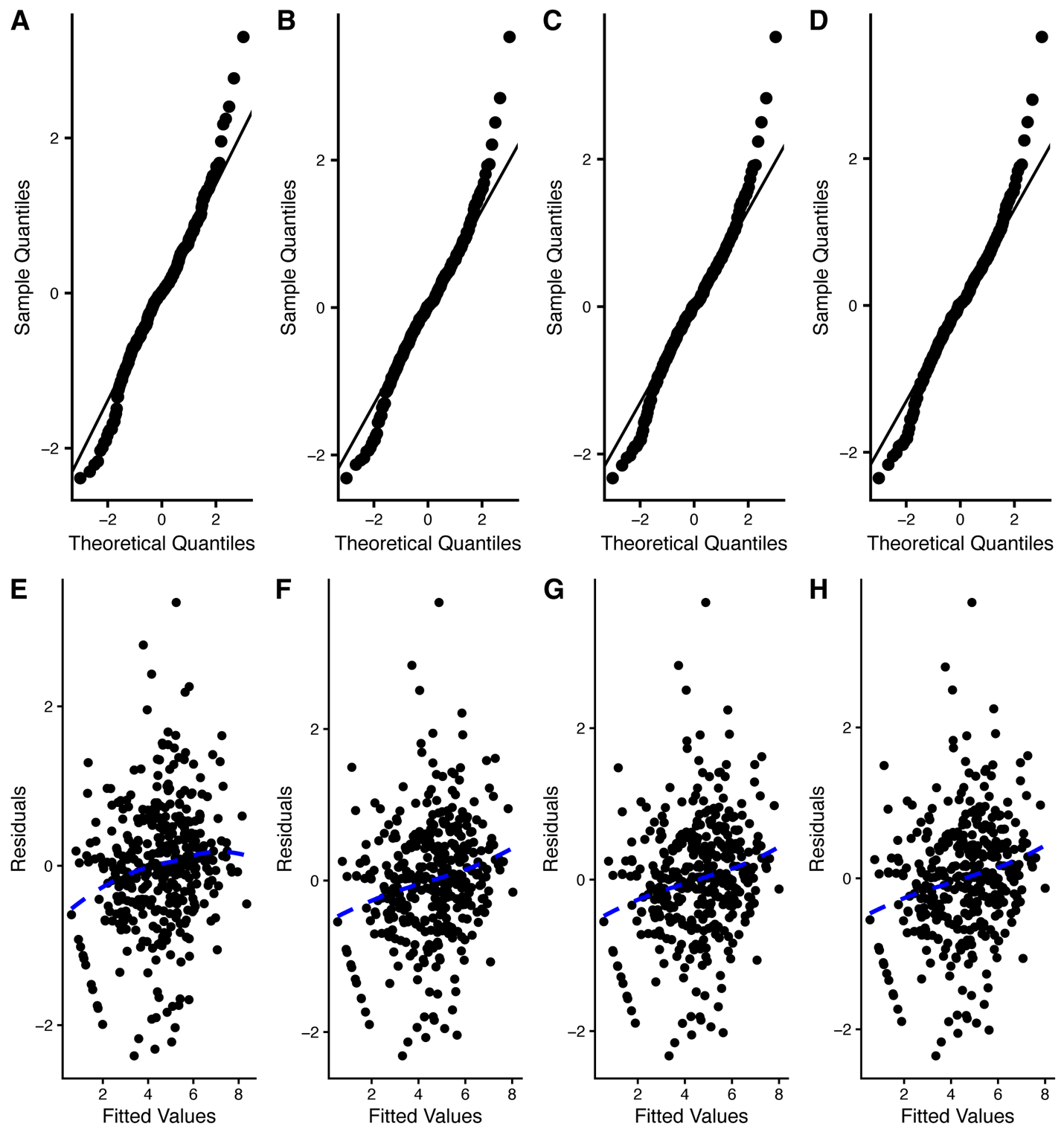


**Supplementary Figure 12**. Q-Q plots and heteroscedasticity plots for LME models predicting depression symptoms across three time points from total lifetime frequency (A, C) and severity (B, D) of five stressor characteristics at baseline assessment, **after** square-root transformation


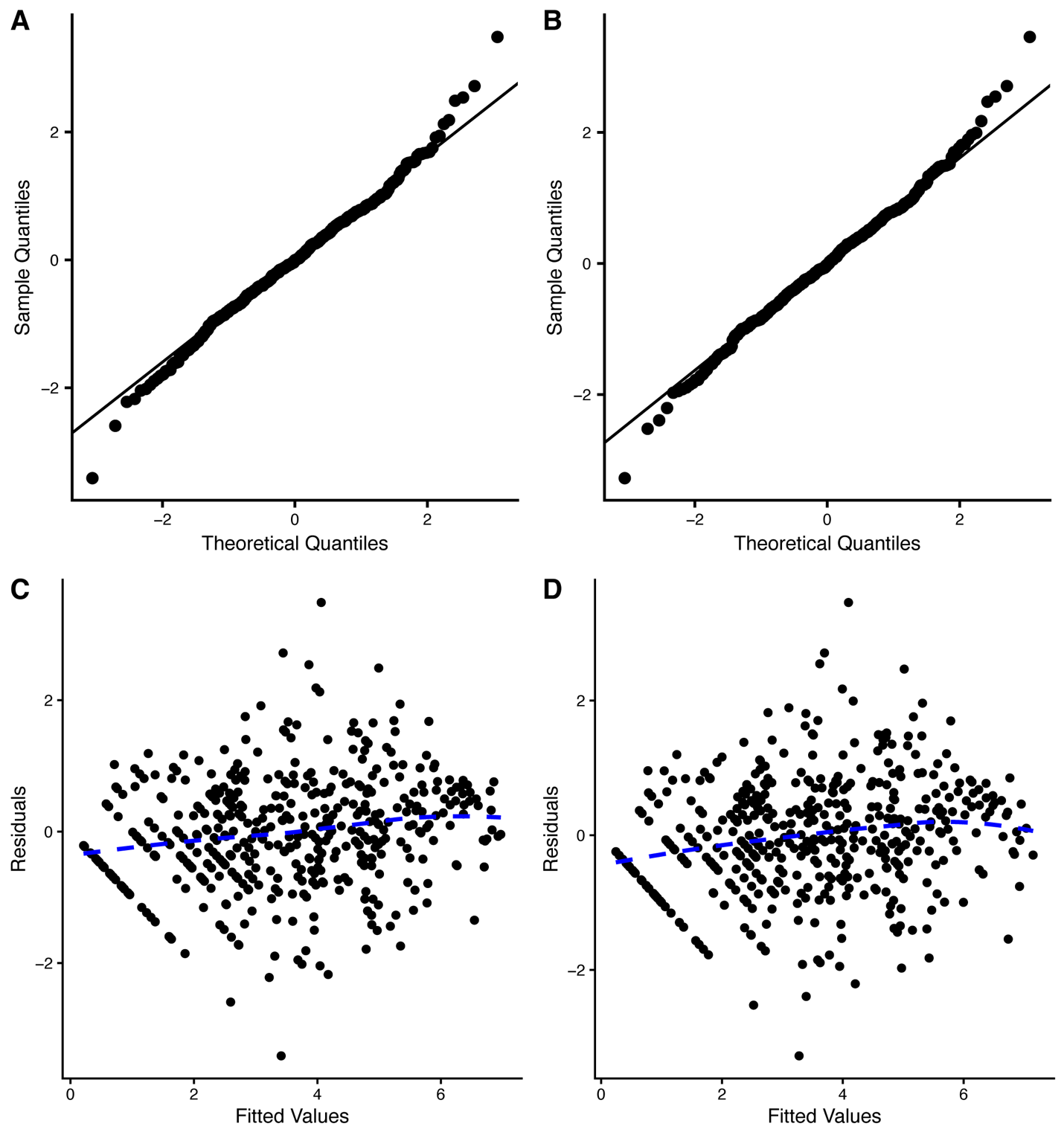


**Supplementary Figure 13**. Q-Q plots and heteroscedasticity plots for LME models predicting depression symptoms across three time points from the four RSFC metrics linked to lifetime entrapment severity at baseline assessment, **after** square-root transformation


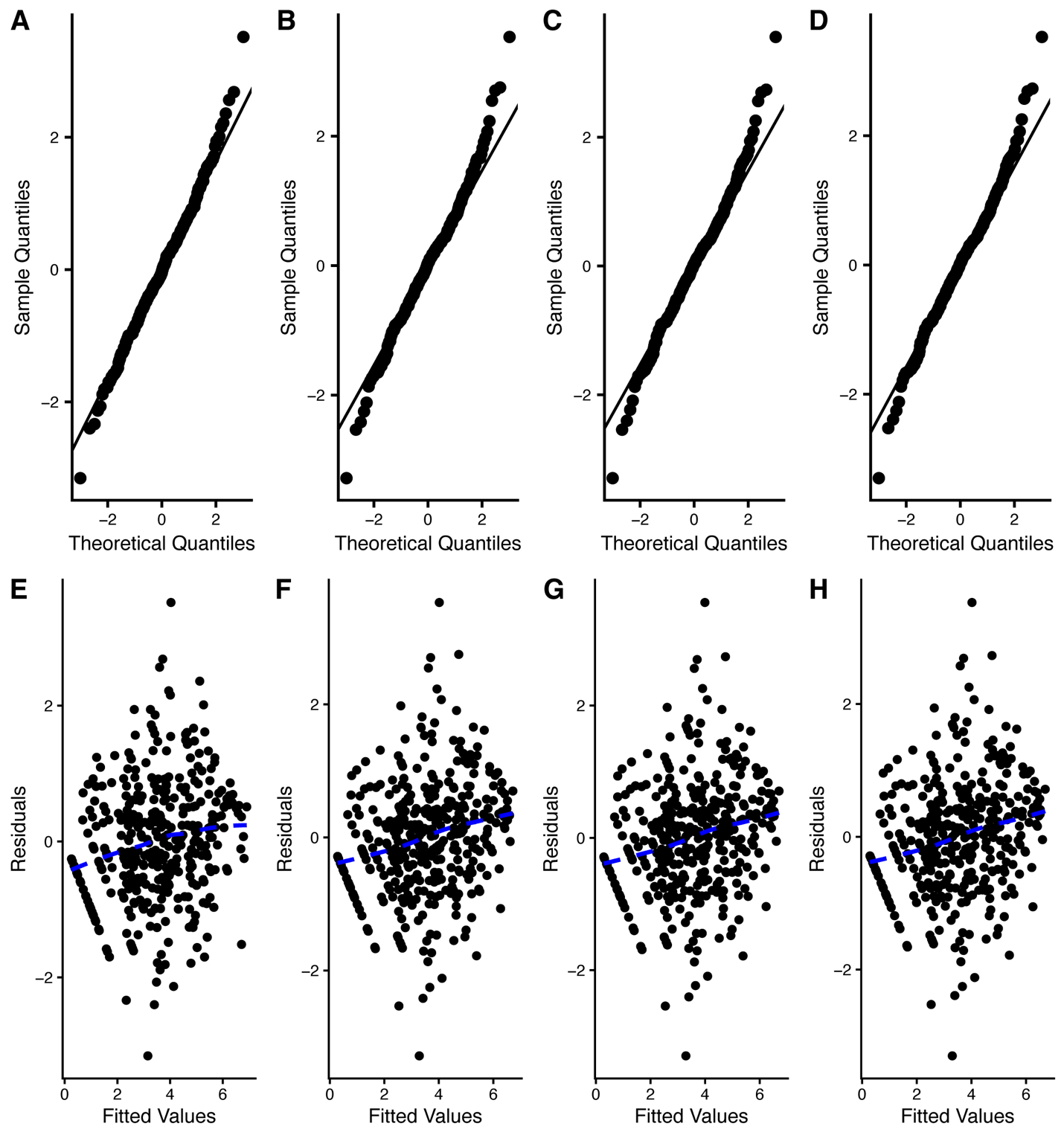

Supplement: Qu et al. supplementary material [file S0033291726103699sup001.docx]
